# Ketogenic diet–induced changes in hepatic drug metabolism with potential implications for ozanimod pharmacokinetics in mice

**DOI:** 10.1371/journal.pone.0357797

**Published:** 2026-09-08

**Authors:** Veronika Frybortova, Stefan Satka, Lenka Jourova, Pavel Anzenbacher, Iveta Zapletalova, Michal Kraus, Klara Kostovcikova, Miloslav Kverka, Eva Anzenbacherova

**Affiliations:** 1 Department of Medical Chemistry and Biochemistry, Faculty of Medicine and Dentistry, Palacky University Olomouc, Olomouc, Czech Republic; 2 Department of Pharmacology, Faculty of Medicine and Dentistry, Palacky University Olomouc, Olomouc, Czech Republic; 3 Laboratory of Cellular and Molecular Immunology, Institute of Microbiology of the Czech Academy of Sciences, Prague, Czech Republic; Universidade do Estado do Rio de Janeiro, BRAZIL

## Abstract

Ketogenic diet (KD) is increasingly considered as an adjunctive therapeutic approach across a range of diseases, but its effects on the pharmacokinetics of concomitantly administered drugs remain unclear. Such interactions may be particularly relevant in multiple sclerosis, where KD is being explored as a complementary strategy alongside disease-modifying therapies, such as ozanimod. We therefore investigated whether KD affects ozanimod metabolism and pharmacokinetics and explored potential factors that may contribute to such effects. Specific pathogen-free female C57BL/6 mice were fed either a control diet containing 10% of calories from fat or a ketogenic diet containing 90% of calories from fat for 4 weeks. Metabolic, inflammatory, and hormonal parameters were determined in plasma. Gut microbiota composition was analyzed by whole-metagenome shotgun sequencing. In parallel, hepatic cytochrome P450 (CYP) enzymes were evaluated by mRNA expression and activity together with ozanimod pharmacokinetics. KD induced the expected metabolic adaptation to ketosis and led to a significant increase in plasma cholesterol accompanied by changes in gut microbiota composition. Other metabolic and inflammatory parameters showed only modest changes. In addition, KD altered the expression and activity of hepatic CYP enzymes, including enzymes involved in ozanimod metabolism: CYP1A activity and mRNA expression were significantly increased in KD-fed mice, whereas lower CYP2C activity was observed in pooled samples and CYP3A activity showed a non-significant trend toward lower values. Ozanimod exposure tended to be higher in KD-fed mice, resulting in an approximately 17% increase in area under the concentration–time curve, although this effect did not reach statistical significance. In conclusion, our findings demonstrate that KD altered the expression and activity of hepatic CYP enzymes and revealed a non-significant trend toward increased ozanimod exposure. These observations highlight the potential importance of considering dietary interventions as a factor contributing to variability in drug response.

## Introduction

The global burden of chronic autoimmune diseases continues to rise, despite major advances in pharmacology, which still primarily provides symptomatic relief rather than addressing the underlying disease mechanisms. Consequently, there is growing interest in exploring novel preventive or adjuvant strategies. One promising approach involves the use of therapeutic diets designed to modulate metabolic pathways, reshape the gut microbiome, influence immune responses, and reduce inflammation [1].

The ketogenic diet (KD) is a nutritional protocol characterized by a very low intake of carbohydrates and a high intake of fats. Originally established in the 1920s as a therapeutic intervention for epilepsy [2], KD has since evolved into a widely used weight-loss strategy and a contemporary lifestyle trend [3]. In recent years, KD has also been explored as a potential adjuvant therapy in several chronic conditions, including metabolic disorders, neurodegenerative diseases, and cancer [4–6]. Experimental studies have begun to uncover underlying mechanisms, suggesting that KD may exert neuroprotective and immunomodulatory effects. In the context of multiple sclerosis (MS), KD may therefore influence two key aspects of disease pathology: chronic inflammation and neurodegeneration. In addition, emerging clinical studies in patients with MS suggest that ketogenic dietary interventions are feasible and well tolerated and may be associated with improvements in quality of life, fatigue, and selected clinical outcomes [7,8].

The range of treatment options for MS is expanding, with more than 15 standard pharmacological therapies currently available [9]. Ozanimod (Zeposia®) is a recently approved sphingosine-1-phosphate receptor modulator indicated for relapsing-remitting MS and ulcerative colitis [10,11]. By selectively targeting S1PR1 and S1PR5, ozanimod modulates immune cell trafficking and thereby reduces autoimmune inflammation [12]. Ozanimod is generally well tolerated and exhibits a distinct pharmacokinetic profile characterized by extensive hepatic metabolism [13]. Its biotransformation involves several cytochrome P450 (CYP) enzymes, particularly CYP1A1, CYP3A4, and CYP2C8, as well as monoamine oxidase B (MAO-B) [14]. Although ozanimod itself exhibits pharmacological activity, its therapeutic efficacy largely depends on the formation of active metabolites generated through hepatic metabolism [15]. These metabolic pathways are essential for both the activation and elimination of the drug and may be modulated by individual-specific factors such as diet, inflammation, or the gut microbiome.

KD has been shown to alter the pharmacokinetics of several drugs [16], raising concerns about its potential to interfere with the metabolism and efficacy of pharmacological treatments. This is particularly relevant in the context of MS, where KD has been suggested as a possible adjunct to standard therapies such as ozanimod.

In addition to its metabolic effects, KD has been associated with increased circulating β-hydroxybutyrate, altered lipid metabolism, hepatic inflammation, and shifts in gut microbiota composition [1,17–19]. Previous studies have shown that these changes may alter hepatic CYP1A, CYP2C, CYP2E1, and CYP3A expression or activity [20–22], suggesting that KD may influence ozanimod biotransformation. Despite the increasing clinical interest in KD, its effects on hepatic enzyme function and xenobiotic metabolism remain insufficiently characterized [23]. Because ozanimod depends on hepatic biotransformation for both elimination and the formation of active metabolites, KD-induced changes in CYP activity, inflammation, or gut microbiota may influence both its systemic exposure and pharmacological activity.

We hypothesized that KD may alter hepatic drug-metabolizing capacity and thereby influence the pharmacokinetics and efficacy of ozanimod. To test this hypothesis, we examined whether KD affects ozanimod pharmacokinetics in mice and explored potential mechanisms underlying this effect, including changes in hepatic CYP expression and activity, inflammatory status, and gut microbiota composition.

## Materials and methods

### Animals

Fifty-two 8- to 10-week-old specific-pathogen-free (SPF) C57BL/6 female mice were used, with an average body weight of 19.6 ± 1.4 g (mean ± SD). The mice were obtained from the breeding colonies of the Institute of Microbiology of the Czech Academy of Sciences. Mice were fed for 4 weeks either a ketogenic diet (KD, D10070801, Research Diets, New Brunswick, NJ, USA) with 90% of calories derived from fat and 10% from protein, or a composition-matched control diet (CD, D19082304, Research Diets, New Brunswick, NJ, USA), provided by the same manufacturer, containing 10% of calories from fat, 80% from carbohydrates, and 10% from protein. All animals were kept in a room with a 12 h light-dark cycle at 22 ± 2 °C. Animals were assigned to experimental groups before the start of the study using weight-based randomization to achieve comparable baseline body weight distribution between groups. No formal blinding procedures were incorporated into the study design.

For pharmacokinetic experiments, ozanimod was diluted in 5% DMSO, 5% Tween 20, and 90% 0.1N HCl, and applied as a single intragastric dose of 5 mg/kg per mouse after 4 weeks on the respective diets. A dose of 5 mg/kg was selected to enable observation of the drug’s pharmacokinetic profile, as this dose had been employed in prior studies where its non-toxic effect was demonstrated [24,25]. At every given time point (0, 2, 4, 6, 8, and 24 hours after ozanimod application), three mice were euthanized and plasma samples were collected.

In subsequent experiments, mice were assigned to each diet group (ketogenic or control) to assess the effects of the ketogenic diet on plasma metabolic parameters, inflammatory and hormonal markers, and the mRNA expression and activity of drug-metabolizing enzymes (8 mice per group).

After 4 weeks on the diets, liver, plasma, and fecal samples were collected. Animals remained on their respective experimental diets *ad libitum* throughout the experiment and no fasting period was implemented prior to euthanasia, blood glucose, ketone, or lipid measurements. Mice were euthanized by isoflurane overdose, followed by cervical dislocation. Blood samples were collected by cardiac puncture through both atria into syringes coated with 0.5 mol/L EDTA (pH 8). Livers, without gallbladders, were removed and stored at −80 °C. Blood plasma was separated by centrifugation (2,500 × g, 15 min, 4 °C), and plasma samples were stored at −80 °C. The experiments were approved by the Committee for the Protection and Use of Experimental Animals of the Institute of Microbiology of the Czech Academy of Sciences (approval ID: 18–2023-P).

### Glucose and β-hydroxybutyrate measurements

Plasma glucose and β-hydroxybutyrate levels were determined at days 0, 1, 2, 3, 5, 7, 10, 12, 14, 21, and 28 from the tail vein using Abbott FreeStyle Optium Neo Blood Glucose and Ketone monitoring system (Abbott Diabetes Care, USA). To minimize stress from frequent blood sampling, a rotational design was applied, in which 2 animals per group were sampled at each individual sampling time, with a minimum of 2 days between consecutive bleeds for each animal. To account for diurnal oscillations in ketone levels driven by natural feeding and fasting cycles [26], measurements were taken 2–4 times daily across both light and dark phases (except on days 0 and 5). For graphical depiction, these intra-day measurements were averaged into daily means.

### Ferric Reducing Antioxidant Power (FRAP) assay

The antioxidant capacity was assessed using the Ferric Reducing Antioxidant Power (FRAP) assay, which is based on the reduction of Fe^3+^ to Fe^2+^ ions under acidic conditions. This reduction leads to the formation of a blue-colored ferrous complex with 2,4,6-tris(2-pyridyl)-1,3,5-triazine, and the increase in absorbance is measured at 593 nm. The assay was performed following the method described previously [27], with ascorbic acid used as the reference standard. The final reaction mixture contained 200 μL of the working FRAP reagent – prepared in a 10:1:1 ratio of 300 mmol/L acetate buffer (pH 3.6), 10 mmol/L TPTZ in 40 mmol/L HCl, and 20 mmol/L FeCl_3_ – and 10 μL of the test sample, standard, or blank. Results were expressed as the molar concentration of ascorbic acid equivalent to the antioxidant activity exhibited by the sample.

### Plasma lipid profile

Total plasma cholesterol was measured using the enzymatic Kit from BioSystems (REF11539). The red-colored product was detected at 510 nm. High-density lipoproteins (HDL) were measured using the same protocol as total cholesterol, with the addition of preliminary steps involving the precipitation of (very)low-density lipoproteins using phosphotungstic acid and Mg^2+^, followed by centrifugation. Due to limited sample volume, pooled samples from each dietary group were used. Triglycerides (TAG) were measured using the enzymatic Kit from BioSystems (REF11529). The colored product was measured photometrically at 510 nm.

### Enzyme-Linked Immunosorbent Assay (ELISA)

Levels of leptin, IL-6, IL-1β, and TNF-α were analyzed in plasma and whole liver homogenates using ELISA murine leptin Kit (900-K76K), ELISA murine IL-6 Kit (900-K50K), ELISA murine IL-1β Kit (900-K47K), and ELISA murine TNF-α Kit (900-K54K) from PeproTech according to the manufacturer’s instructions. Due to limited sample volume, pooled samples from each dietary group were used.

### Determination of ozanimod in murine plasma

The pharmacokinetic profile of ozanimod was determined using a previously validated HPLC method with fluorescent detection [28]. Briefly, mouse plasma was mixed with internal standard, nabumetone, precipitated with acetonitrile containing 0.1% HCl, centrifuged, evaporated under nitrogen flow at 40 °C, and finally reconstituted in 100 µL of mobile phase.

HPLC studies were performed using the Shimadzu LC-20 HPLC system (Shimadzu, Kyoto, Japan) equipped with UV/fluorescence detection. Separation was achieved on a Chromolith HighResolution RP-18e monolithic column (100 × 4.6 mm; Merck, Darmstadt, Germany), fitted with a HighResolution RP-18 endcapped guard column (5 × 4.6 mm; Merck, Darmstadt, Germany). The mobile phase consisted of 16 mmol/L sodium acetate (pH 4.7) and acetonitrile (1.7/1; v/v). Data were analyzed using LabSolutions software (Shimadzu, Kyoto, Japan).

### Gene expression analysis

Total RNA was isolated from murine liver tissues stored in RNA later using an RNeasy Mini Kit (Qiagen, Hilden, Germany). The concentration and purity of total RNA were quantified spectrophotometrically using the NanoPhotometer® N60 (Implen, Munich, Germany). 1000 ng of RNA was converted to single-stranded cDNA using the Transcriptor High Fidelity cDNA Synthesis Kit (Roche, Basel, Switzerland). The real-time qPCR was performed on the LightCycler 1536 Instrument (Roche) using commercial TaqMan Gene primers, shown in Table 1 (Thermo Fisher Scientific, Waltham, MA, USA). The 1536-well plates were pipetted using the Echo Liquid Handler (Labcyte, Dublin, Ireland). The mRNA expressions were calculated using the 2^(-ΔΔC(T))^ method [29], and the values of target genes were normalized to the values for the housekeeping gene hypoxanthine guanine phosphoribosyl transferase (*Hprt*). The data from gene expression analysis are available online at Zenodo: https://doi.org/10.5281/zenodo.17250497.

**Table 1:** TaqMan Gene Expression Assays (Thermo Fisher Scientific).

Gene	Assay ID
*Hprt* (housekeeping)	Mm03024075_m1
*Cyp1a2*	Mm00487224_m1
*Cyp2a5*	Mm00487248_g1
*Cyp2b10*	Mm00456591_m1
*Cyp2c29*	Mm00725580_s1
*Cyp2c38*	Mm00658527_m1
*Cyp2d22*	Mm00530542_m1
*Cyp2e1*	Mm00491127_m1
*Cyp3a11*	Mm00731567_m1
*Cyp3a13*	Mm00484110_m1
*Ahr*	Mm00478932_m1
*Hmgcs2*	Mm00550050_m1
*Hmgcl*	Mm00500497_m1

### Liver microsomal fractions and cytochrome P450 enzyme activity assays

Every microsomal fraction was prepared from the whole mouse liver by differential centrifugation according to the established protocol [30] and then stored at −80 °C. Concentrations of CYP enzymes were determined spectrophotometrically using carbon monoxide [31], and total protein content was measured using a bicinchoninic acid assay from Thermo Fisher Scientific (cat: 23228). The activities of enzymes, orthologues to human CYPs, were measured in liver microsomal fractions according to the established methods [31,32]. Generally, individual incubation mixtures contain potassium phosphate buffer (pH 7.4), NADPH-generating system (NADP^+^, isocitrate, isocitrate dehydrogenase and MgCl_2_), liver microsomes and the specific substrates: ethoxyresorufin (CYP1A); coumarin (CYP2A), 7-ethoxy-4-trifluoromethylcoumarin (CYP2B), diclofenac (CYP2C), bufuralol (CYP2D), chlorzoxazone (CYP2E) and diazepam (CYP3A). For the determination of metabolites, a Shimadzu LC-20 HPLC system with UV or fluorescence detection was used. The analyses were performed with a LiChrospher RP-18 column (5 μm), 250 × 4 mm (Merck).

### Gut microbiota analysis

Fecal samples were collected after 4 weeks of dietary intervention and placed in company-provided tubes containing preservative solution, then sent to TransnetYX (Cordova, TN, USA) for DNA extraction, library preparation, and shotgun whole-metagenome sequencing using Illumina NextSeq 2000 instrument at a sequencing depth of 2 million 2x150 bp read pairs per sample. The data of composition of the gut microbiome, species relative abundances, alpha and beta diversity were analyzed using the One Codex platform (One Codex, San Francisco, CA, USA). Detailed information regarding the proprietary read quality filtering, taxonomic assignment criteria, and species-level abundance estimations can be found at: https://docs.onecodex.com/en/articles/6891294-transnetyx-sequencing-and-analysis-methods. Raw sequencing data are available at https://www.ncbi.nlm.nih.gov/sra/PRJNA1336998.

### Plotting and statistical analysis

The data were analyzed using GraphPad Prism 8.4.3 (GraphPad Software, Inc.) and RStudio 2025.05.1 Build 513 (RStudio, Inc.). Due to the small sample size (n = 8), non-parametric tests were used for statistical evaluation, specifically the Mann-Whitney U test. For analyses involving simultaneous evaluation of multiple parameters, a correction for multiple comparisons using the Benjamini, Krieger, and Yekutieli was applied, and differences were considered statistically significant at q < 0.05. Longitudinal blood glucose and β-hydroxybutyrate data were analyzed using a two-way mixed-effects model with REML estimation and Geisser-Greenhouse correction; significance is reported for the diet factor. Statistical evaluation of microbiome diversity metrics and differential taxonomic abundances was performed using Mann-Whitney U test, PERMANOVA or multiple t-test with Holm-Sidak correction for multiple comparisons. Pharmacokinetic AUC values were calculated by the trapezoidal rule and compared between groups using Bailer’s method, appropriate for destructive sampling designs, with a two-sided t-test and Welch-Satterthwaite degrees of freedom. Details of the statistical tests used are provided in the figure legends.

## Results

### Ketogenic diet induces metabolic adaptation

**Fig 1:**
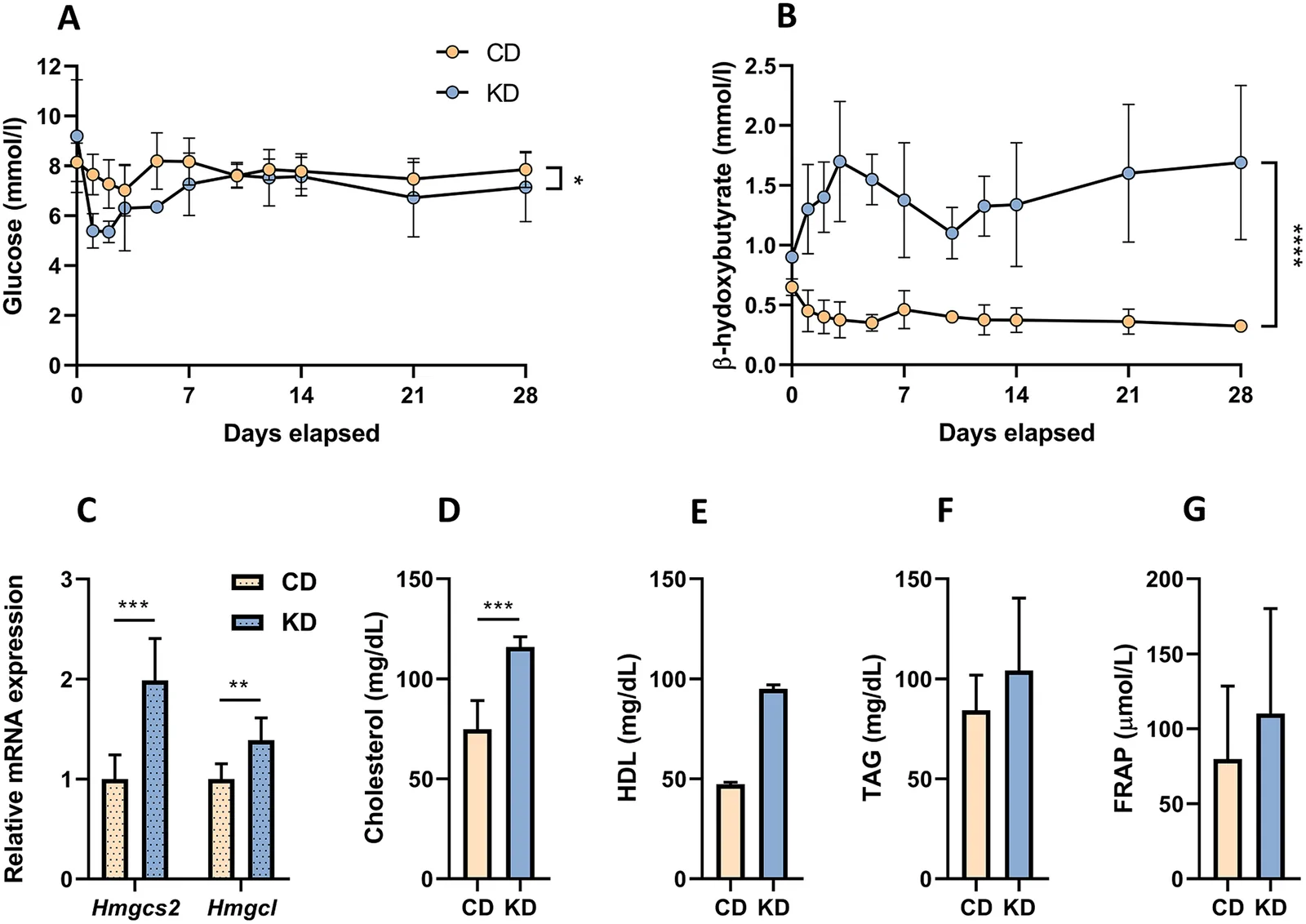
Effect of ketogenic diet on plasma metabolic parameters. A, glucose levels; B, β-hydroxybutyrate levels. Data are depicted as daily means ± SD (n = 2–8); Statistical analysis was performed using a two-way mixed-effects model (REML, Geisser-Greenhouse correction); asterisks indicate the effect of diet; C, relative mRNA expression; D, total cholesterol; E, high-density lipoproteins (measured using pooled plasma samples, error bars represent ± SD of technical triplicates); F, triglycerides; G, total antioxidant capacity (measured by the FRAP assay). Data are presented as a mean ± SD **(n** = **8).** Statistical analysis was performed using the Mann-Whitney U test with * P < 0.05; ** P < 0.01; *** P < 0.001; **** P < 0.0001.

As an initial step, we evaluated selected metabolic parameters to confirm metabolic adaptation to KD. While the glucose levels dropped, particularly within the first week (Fig 1A), the concentration of circulating BHB increased significantly throughout the entire study period, as expected under KD conditions (Fig 1B). In the livers of KD-fed mice, the mRNA levels of 3-hydroxy-3-methylglutaryl-CoA synthase 2 (*Hmgcs2*) and 3-hydroxy-3-methylglutaryl-CoA lyase (*Hmgcl*) were significantly elevated, reflecting the diet’s impact on ketone body synthesis (Fig 1C). Altogether, these changes indicate a metabolic shift toward ketogenesis [17].

Total plasma cholesterol was increased in KD-fed mice compared to CD (Fig 1D). Higher HDL levels were observed in pooled plasma samples from KD-fed mice (Fig 1E). Plasma TAG concentration was not affected by KD (Fig 1F). Total antioxidant capacity measured by the ferric reducing antioxidant power (FRAP) assay was slightly increased in KD-fed mice compared to CD-fed animals, although the difference was not statistically significant (Fig 1G). This trend aligns with previous findings suggesting that a KD may improve redox status, for example, by increasing mitochondrial glutathione levels in the liver [33]. However, the FRAP assay primarily reflects overall ferric-reducing capacity and therefore does not specifically assess mitochondrial redox balance or the activity of antioxidant enzymes.

### Ketogenic diet modulates hepatic inflammatory markers

**Fig 2:**
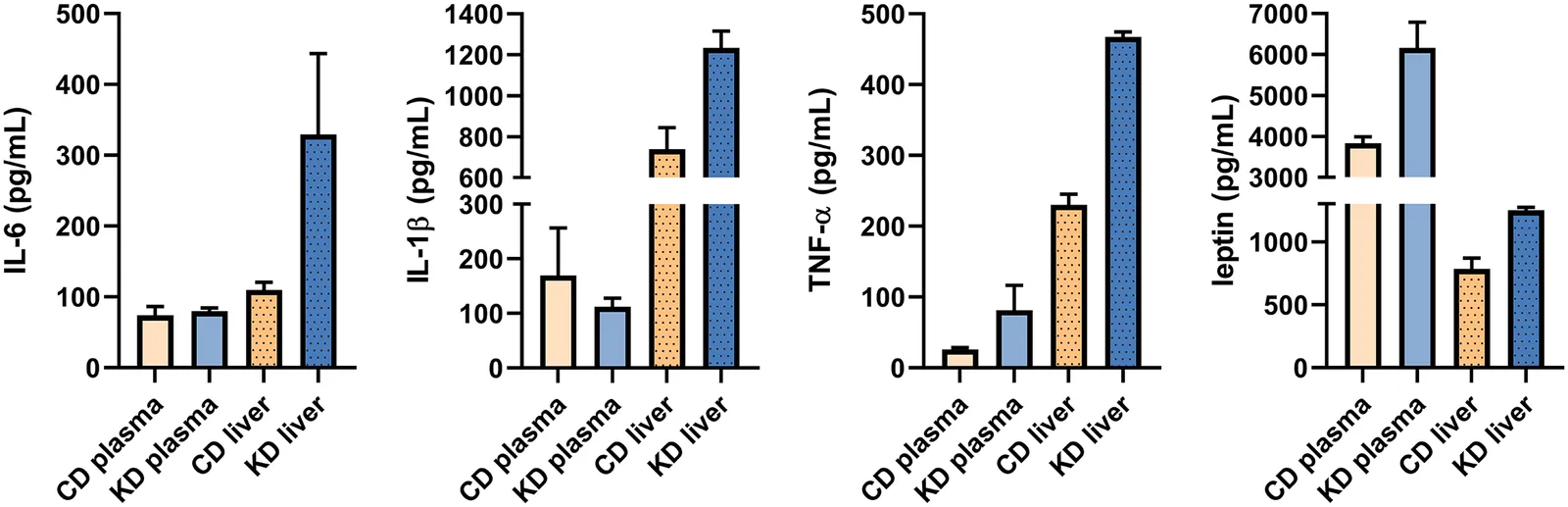
Effect of ketogenic diet on pro-inflammatory cytokine and leptin levels in plasma and livers. Measured using pooled plasma or liver homogenate samples. Data are presented as a mean, and error bars represent ± SD of technical triplicates.

To further explore systemic effects of the KD, we measured circulating levels of pro-inflammatory cytokines and leptin in pooled plasma and liver homogenate samples using ELISA. Higher levels of IL-6, IL-1β, and TNF-α were observed in liver homogenates from KD-fed mice compared to CD-fed animals. In plasma, higher TNF-α levels were observed, whereas IL-6 and IL-1β levels appeared comparable between dietary groups. Higher leptin levels were also observed in plasma and liver from KD-fed mice (Fig 2). This outcome was somewhat unexpected, as KD is often associated with reduced fat mass and lower circulating leptin levels [34]. Together, these metabolic, hormonal, and inflammatory observations provide additional context for the hepatic adaptations induced by KD. Because inflammatory markers and leptin were assessed in pooled samples, these findings should be interpreted with caution.

### Ketogenic diet reduces alpha diversity

**Fig 3:**
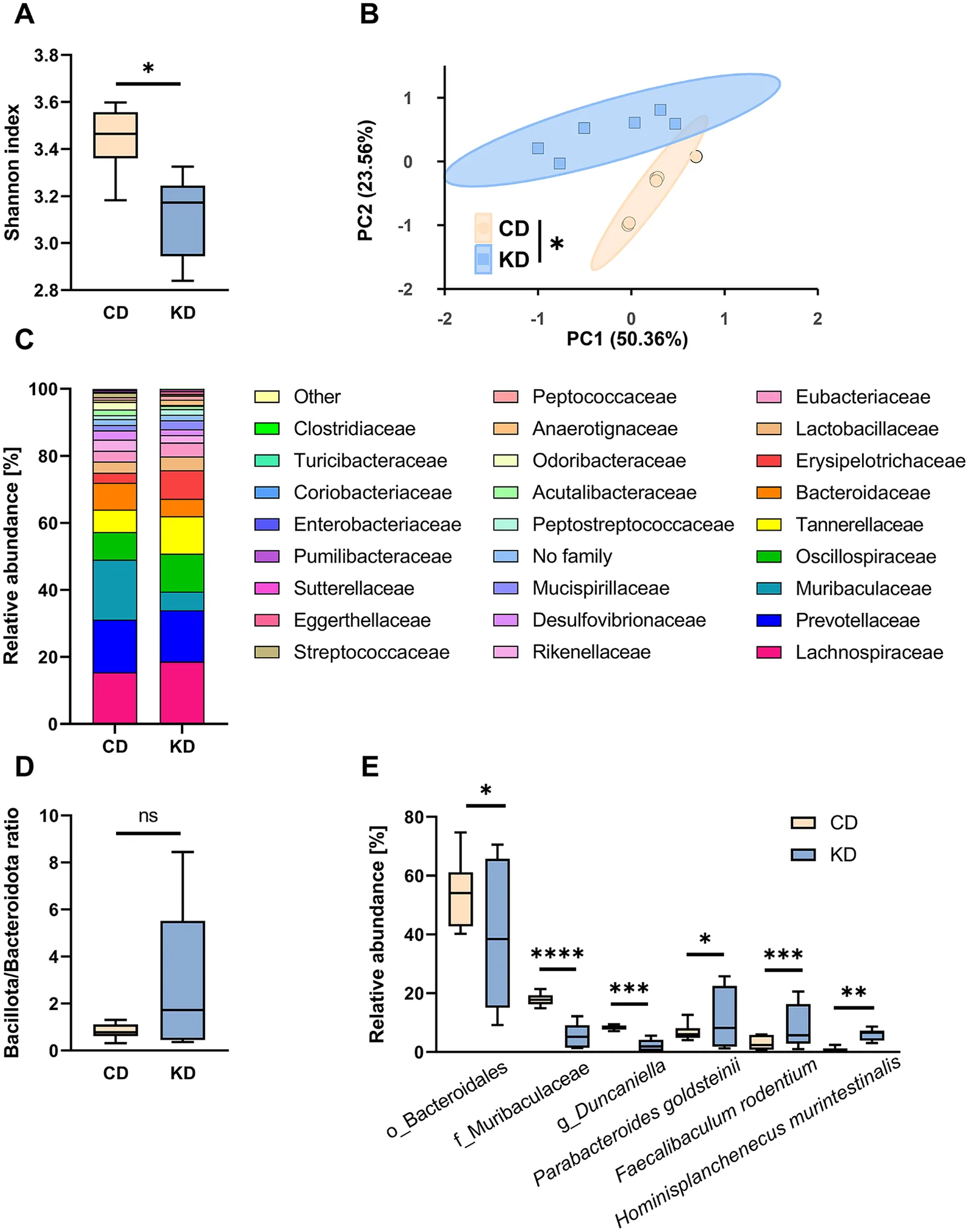
Ketogenic diet induced gut microbiota changes. A, Shannon index. Statistical analysis was performed using the Mann-Whitney U test; B, principal coordinate analysis (PCoA) based on Bray-Curtis dissimilarity with 95% confidence ellipses for each group. Statistical significance was assessed using PERMANOVA; C, mean relative abundances of observed families; D, Bacillota to Bacteroidota ratio. Statistical analysis was performed using the Mann-Whitney U test; E, relative abundances of significantly changed strains. Statistical analysis was performed using multiple t-tests with Holm-Sidak correction for multiple comparisons. * P < 0.05; ** P < 0.01; *** P < 0.001; **** P < 0.0001.

To compare the effects of KD and CD on gut microbiota composition, we collected fecal samples after 4 weeks on either diet and analyzed them by shotgun whole-metagenome sequencing. The KD group showed significantly lower alpha diversity represented by Shannon index (Fig 3A). The beta diversity analysis based on the differences in bacterial abundances showed significant shifts between KD and CD groups (Fig 3B, 3C). Based on the relative abundances, a marked but non-significant increase in the Bacillota/Bacteroidota ratio was found (Fig 3D). Several bacterial taxa were differentially abundant between KD and CD groups (Fig 3E).

### Ketogenic diet influences liver enzyme activities involved in drug metabolism

To investigate whether KD influences hepatic drug-metabolizing capacity, we measured activities of selected cytochrome P450 (CYP) enzymes in liver samples from mice fed either KD or CD. These enzymes were chosen for their central role in the metabolism of a wide range of clinically used drugs – accounting for approximately 80% of all therapeutic agents, including ozanimod [14].

**Fig 4:**
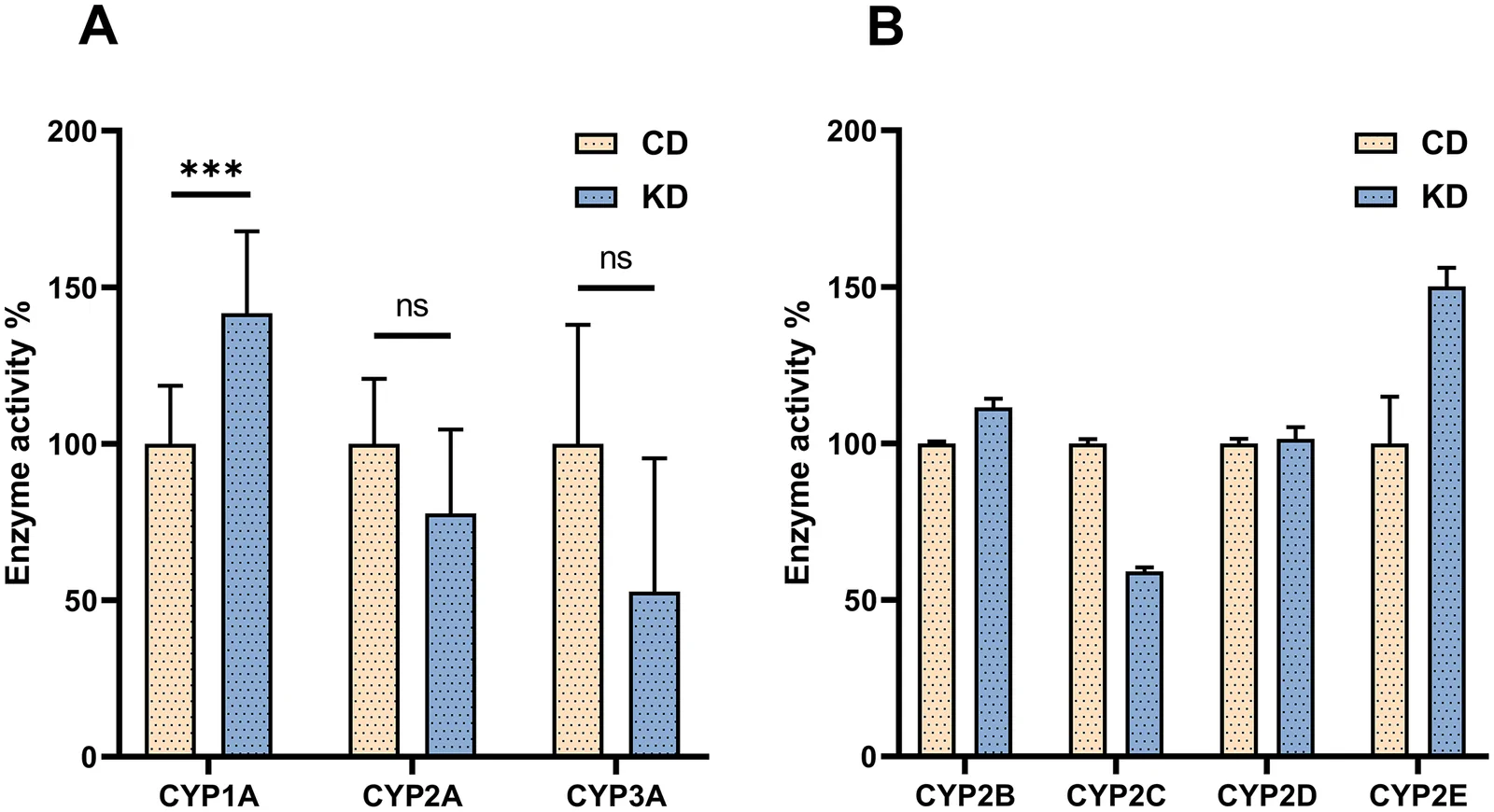
Enzyme activity of selected hepatic microsomal cytochromes P450 (CYP). A, enzyme activity measured individually in each animal (n = 8). Data are expressed as mean ± SD. The statistical analysis was performed using the Mann-Whitney U test with *** P < 0.001; B, enzyme activity measured in pooled microsomal samples (pools of 8 mice per group), due to limited liver tissue and high enzyme demands of the assay; therefore, statistical analysis was not performed. Data are expressed as mean ± SD of technical triplicates.

Mice fed KD exhibited a significant increase (41%) in hepatic CYP1A activity compared to CD-fed mice (Fig 4A). Although hepatic CYP3A activity tended to be lower (approximately 47% reduction) in KD-fed mice compared to CD-fed mice, this difference did not reach statistical significance due to high inter-individual variability. Because CYP2E and CYP2C activities were determined in pooled microsomal samples due to limited tissue availability, statistical analysis was not feasible. Higher CYP2E activity and lower CYP2C activity were observed in KD-fed mice compared with CD-fed mice (Fig. 4B).

### Ketogenic diet alters hepatic gene expression of cytochrome P450

Given the observed changes in hepatic CYP enzyme activities associated with KD, we next examined whether these alterations were accompanied by transcriptional regulation. To this end, hepatic mRNA expression levels of key genes encoding CYP enzymes involved in drug metabolism, including those implicated in ozanimod biotransformation, were assessed [14].

**Fig 5:**
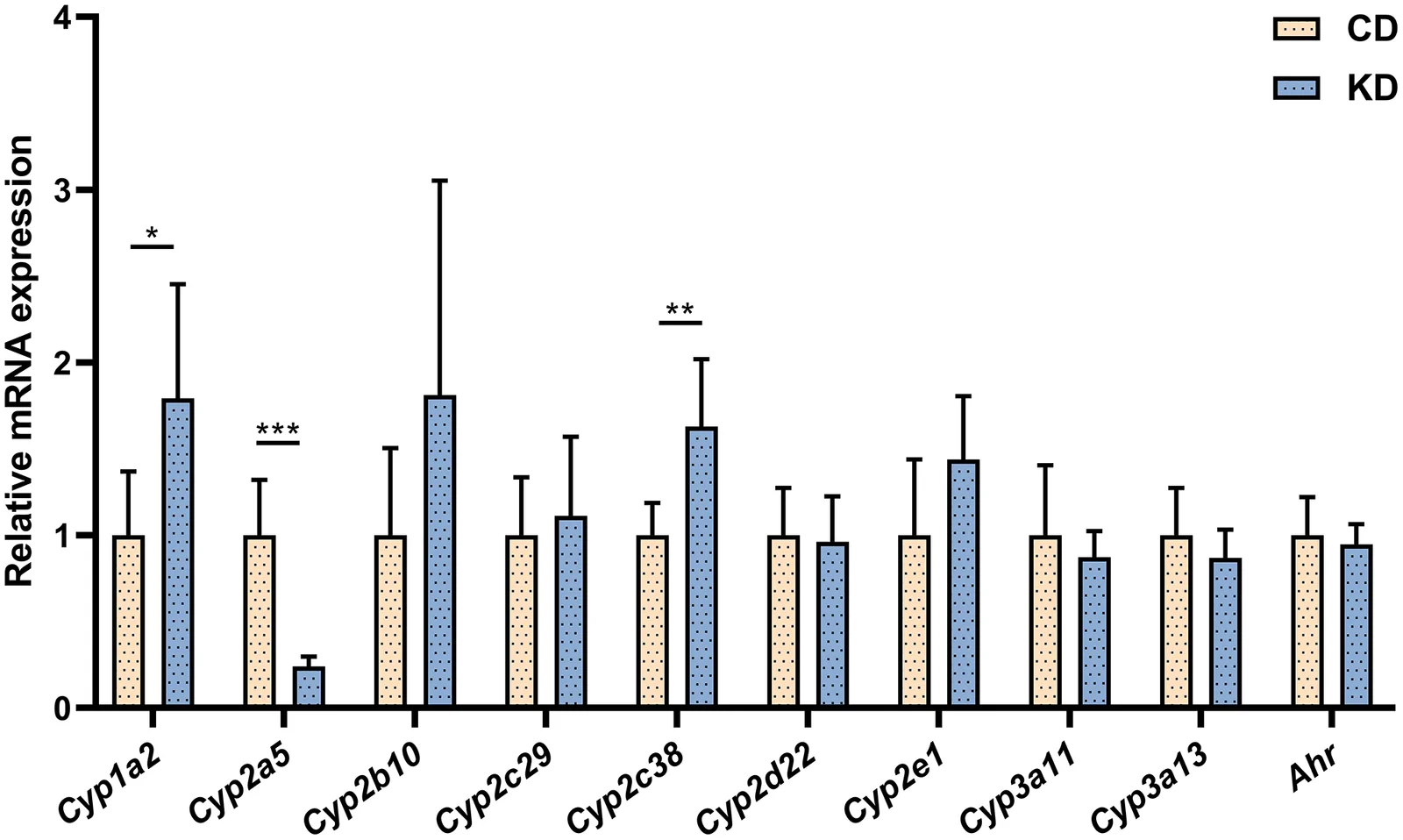
Hepatic mRNA expression of cytochrome P450 (*Cyp*) genes. The mRNA expression levels of *Cyp* genes were measured in liver tissue from mice. Gene expression was assessed individually in each animal (n = 8 per group) using quantitative RT-PCR and normalized to the reference gene *Hprt*. Data are expressed as mean ± SD **(n** = **8).** The statistical analysis was performed using the Mann-Whitney U test with * P < 0.05; ** P < 0.01; *** P < 0.001.

Consistent with the enzymatic activity data, hepatic mRNA expression of *Cyp1a2* was significantly upregulated in KD-fed mice compared to CD-fed mice (Fig 5). In addition, *Cyp2c38* expression was also markedly elevated in the KD group. On the other hand, decreased mRNA levels were detected in the case of *Cyp2a5*, suggesting diet-induced transcriptional modulation of specific drug-metabolizing enzymes. Furthermore, we included the aryl hydrocarbon receptor (Ahr), a transcription factor known to regulate the *Cyp1a* subfamily. No significant changes were observed in the mRNA expression of *Ahr*, indicating that *Cyp1a2* upregulation may occur through mechanisms other than transcriptional regulation of Ahr, potentially via Ahr ligand activation.

### Ketogenic diet shows a trend towards increased ozanimod plasma exposure in mice

**Fig 6:**
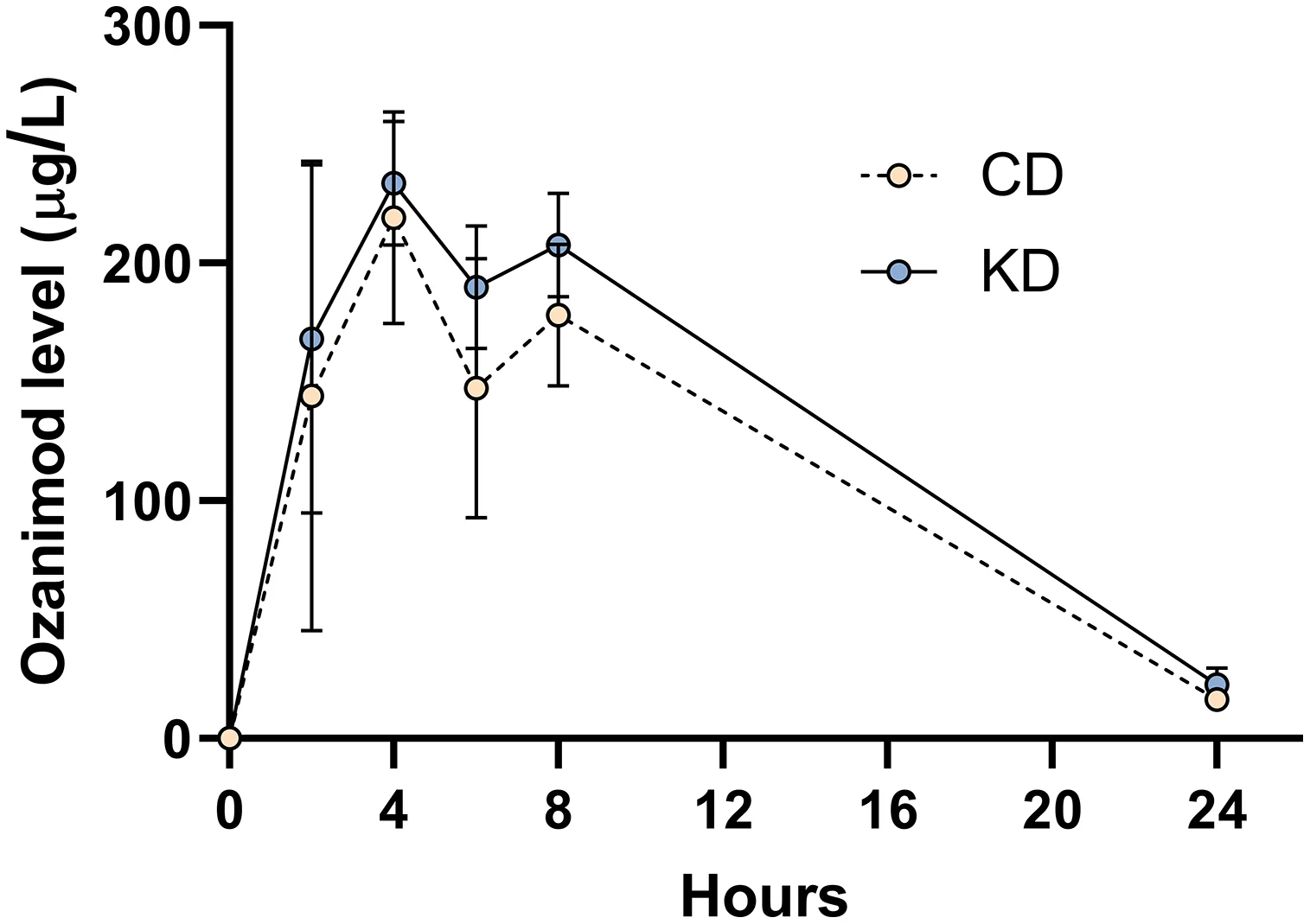
Levels of ozanimod in the plasma of mice fed by KD or CD for 4 weeks. Plasma levels of ozanimod were determined 2, 4, 6, 8, and 24 hours after administration to mice (the 0 hour taken as a control). Data are expressed as mean ± SD with 3 mice per timepoint. Statistical analysis was performed using Bailer’s method for destructive sampling.

In this study, we assessed the pharmacokinetics of ozanimod in mice fed either KD or CD. This experimental design enabled the assessment of potential dietary effects on ozanimod plasma levels. The time to reach the maximum plasma concentration of ozanimod (t_max_) was identical in both dietary groups, occurring at 4 hours post-administration. Higher plasma ozanimod concentrations were observed in KD-fed mice than in CD-fed mice at all measured time points (Fig 6). Accordingly, KD-fed mice exhibited a 17.3% higher area under the plasma concentration–time curve (AUC0–24) than CD-fed mice, representing a non-significant trend (p = 0.138).

## Discussion

Although current therapeutic strategies for multiple sclerosis (MS) are effective to some extent, treatment responses vary considerably among patients [35] due to the multifactorial nature of MS, encompassing genetic, environmental, and immunological variables [36]. This variability in treatment response is strongly influenced by established risk factors such as age at onset, sex, body mass index (BMI), and gut microbiome composition, several of which may themselves be modulated by diet. Notably, higher BMI and altered microbiome profiles have been linked to earlier symptom onset and greater disease severity, both recognized predictors of poor therapeutic response in MS [37]. Variability in treatment response, combined with treatment-related risks like infections, cardiovascular issues, and liver toxicity, has driven growing patient interest in alternative approaches to enhance quality of life [38]. Among these, dietary interventions, such as ketogenic diets (KD), as well as supplementation and lifestyle modifications, have gained considerable attention [7]. The KD has been investigated for neuroprotective and anti-inflammatory properties, but much less is known about how such dietary interventions may influence the pharmacokinetics of disease-modifying therapies and symptomatic treatments in MS.

Because ozanimod undergoes extensive hepatic biotransformation and may be used together with KD in clinical practice, we aimed to investigate whether concomitant exposure to KD may influence ozanimod metabolism and pharmacokinetics. To address this question, we evaluated KD-associated metabolic and molecular changes, including alterations in plasma lipid profile, inflammatory markers, gut microbiota composition, and hepatic cytochromes P450 (CYP), as potential pathways that may contribute to such effects.

As expected, KD induced metabolic adaptation, as reflected by increased β-hydroxybutyrate concentration and elevated hepatic *Hmgcs2* and *Hmgcl* expression, and was accompanied by changes in the lipid profile. In particular, total cholesterol concentrations were increased, whereas HDL showed an increasing trend and TAG levels were unaffected. The impact of KD on lipid metabolism remains controversial, with reports of both beneficial effects, such as improved insulin sensitivity and increased HDL, and unfavorable changes including hypercholesterolemia [1,39,40]. Consistent with our findings, previous studies in mice have also reported elevated cholesterol levels following KD intervention [18,19]. These discrepancies may partly reflect differences in dietary composition and duration of intervention, as well as the distinct metabolic context of healthy and disease models. Similarly, the effects of KD on inflammatory status remain controversial and appear to depend on the experimental and metabolic context, including dietary composition and intervention conditions [19,41–45]. The observed differences in pro-inflammatory cytokines, particularly IL-6, IL-1β, and TNF-α, may reflect modulation of selected inflammatory pathways under the present experimental conditions. However, because these markers were assessed in pooled samples and liver histology was not performed, no conclusions can be drawn regarding the overall hepatic inflammatory status or liver injury.

Nowadays, dietary interventions have emerged as promising adjunctive therapeutic approaches across a range of diseases due to their ability to modulate systemic metabolism and physiological responses [46]. Beyond their direct effects on disease-related pathways, such interventions may also influence factors contributing to variability in drug response and pharmacokinetics, potentially affecting the efficacy and safety of concomitantly administered drugs. Therefore, we assessed the effect of KD on hepatic drug-metabolizing enzymes, with a particular focus on cytochrome P450 (CYP), which plays a major role in ozanimod biotransformation [14]. The ability of dietary lipids and KD to modulate CYP regulation has been previously reported [47–49]. In our study, KD was associated with altered activity of selected CYP enzymes, including enzymes involved in ozanimod metabolism (Fig 4A, 4B). Among these, we observed a trend toward lower activity of CYP2C and CYP3A compared with CD-fed mice. Because of limited sample availability, activity of selected enzymes, including CYP2C and CYP2E, was determined using pooled samples, which precluded assessment of inter-individual variability for these parameters. In selected cases, these findings were further supported by corresponding alterations at the mRNA level. This was particularly evident for *Cyp1a2*, where increased mRNA expression corresponded to elevated CYP1A activity, consistent with a previous study reporting a 213% increase in hepatic *Cyp1a2* expression in KD-fed mice [20]. At the same time, no changes were detected in mRNA expression of *Ahr*, suggesting that *Cyp1a2* induction may result from receptor activation by ligands rather than changes in its transcriptional levels [21]. In contrast, reduced *Cyp1a2* expression has previously been reported in mice fed KD, although this effect was not significant in healthy liver and became more pronounced in the presence of liver fibrosis [18]. *Cyp2a5* expression was significantly reduced despite no apparent change in enzymatic activity, suggesting that transcriptional changes may not directly translate into functional enzyme output and could reflect additional layers of regulation, including post-transcriptional or post-translational mechanisms.

The precise mechanisms responsible for the observed changes in CYP expression and activity are difficult to determine from the present data. However, several possible explanations can be hypothesized, including regulation by microbial metabolites, endogenous metabolites generated during ketosis, and inflammatory signaling. In the present study, KD was associated with altered gut microbiota composition, consistent with previous reports in KD-fed mice [1]. Such changes may lead to altered production of microbial metabolites, which can act as ligands for transcription factors involved in CYP regulation, including AhR [21]. More broadly, both diet- and microbiota-derived metabolites are increasingly recognized as signaling molecules that extend beyond their role as energy substrates and may influence gene expression and metabolic regulation in peripheral tissues [50]. Our previous work further demonstrated that gut microbiota-derived metabolites may influence hepatic drug metabolism and pharmacokinetics [21,51,52]. However, the precise mechanisms by which microbial metabolites might influence ozanimod metabolism were not investigated in the present study. Likewise, endogenous metabolites generated during ketosis, such as β-hydroxybutyrate (BHB), may influence gene expression through epigenetic mechanisms, including inhibition of histone deacetylases and serving as substrates for histone β-hydroxybutyrylation [53].

**Taken together, the metabolic, inflammatory, microbiota-related, and** CYP **changes observed in** KD**-fed mice raise the possibility that** KD **may influence hepatic drug disposition through multiple interacting mechanisms. We therefore examined whether these alterations were accompanied by changes in the pharmacokinetic profile of ozanimod, which served here as a model drug because its metabolism depends strongly on hepatic biotransformation.**

**We found that** KD**-fed mice showed consistently higher plasma concentrations of ozanimod throughout the experiment, resulting in an approximately 17% increase in** AUC **compared with the** CD **group.** However, this difference did not reach statistical significance and should therefore be interpreted with caution. The pharmacokinetic analysis was based on a limited number of animals per time point, which may have contributed to the variability of the results and may partly explain why the increase in ozanimod exposure did not reach statistical significance. Nevertheless, the observed trend is consistent with previous reports indicating that KD may alter the pharmacokinetics of various drugs [16]. At first glance, the higher plasma concentrations of ozanimod might be attributed to the high fat content of KD, since lipophilic drugs may exhibit altered absorption under such conditions [48]. However, clinical data indicate that ozanimod pharmacokinetics are not substantially affected by food intake or dietary fat [13]. Therefore, the observed trend may be related to long-term metabolic adaptation induced by KD, including altered hepatic enzyme activity and other metabolic changes associated with KD. The observed trend toward higher plasma concentrations of ozanimod is consistent with the reduced activity of CYP2C and CYP3A observed in KD-fed mice [14]. Because ozanimod exerts most of its pharmacological activity through active metabolites, reduced CYP activity could also limit their formation and thereby potentially diminish the overall therapeutic effect. Because only the parent compound ozanimod was measured, the overall functional consequence of these changes cannot be determined from the present study. Although KD-induced changes in gut microbiota composition, inflammatory status, and hepatic CYP activity were observed, the causal relationships among these factors remain speculative. Thus, the observed pharmacokinetic trend may indicate an effect of KD on ozanimod exposure, while its consequences for active metabolite formation and pharmacological activity remain unclear. Further studies are needed to determine the relative contribution of individual mechanisms and to establish whether similar changes occur during long-term treatment or in disease models that more closely resemble clinical conditions. Overall, our findings show that KD alters hepatic CYP expression and activity, including enzymes involved in ozanimod metabolism, and reveal a preliminary, non-significant trend toward higher ozanimod exposure. These findings support dietary interventions as a factor that may contribute to variability in drug metabolism and pharmacokinetics, warranting confirmation in larger, targeted pharmacokinetic studies.
