# Supplementary material for: Ketogenic diet–induced changes in hepatic drug metabolism with potential implications for ozanimod pharmacokinetics in mice
Source: PLoS One. 2026 Sep 8;21(9):e0357797. doi: 10.1371/journal.pone.0357797 (PMC13552735; doi:10.1371/journal.pone.0357797)
Supplement: Supplementary file 1 [file pone.0357797.s002.docx]

**Reviewer #1: The present study is highly relevant to the scientific community, given the considerable increase in the association of a ketogenic diet with neurological and neurodegenerative diseases. It is essential for studies to evaluate the effect of this dietary pattern on drugs widely used in the treatment of these diseases.
However, there are some points that require revision and a few concerns that should be addressed.**

We sincerely thank Reviewer #1 for the careful evaluation of our manuscript and for the constructive comments and suggestions. We appreciate the positive assessment of the relevance of our study and believe that the reviewer’s comments helped improve the clarity and quality of the manuscript. Our point-by-point responses are provided below.

**1. Why did you use female subjects, given that they can influence hormone levels?**

Female mice were selected intentionally to increase the translational relevance of the study, as multiple sclerosis occurs more frequently in women (Compston and Coles. Lancet. 2008 doi:10.1016/S0140-6736(08)61620-7) and ozanimod is also administered to female patients. We acknowledge that hormonal fluctuations may influence hepatic metabolism, inflammatory responses, and pharmacokinetics. Future studies may further address the potential contribution of reproductive status and evaluate possible sex-specific responses.

**2. Why was the experiment conducted over only 4 weeks? According to some studies, the ideal approach for analyzing the progression of neurodegenerative diseases is long-term (chronic). (Line 80)**

The duration of 4 weeks was selected intentionally because the aim of this study was not to model the long-term progression of neurodegenerative disease, but to perform an initial assessment of whether ketogenic diet may influence hepatic drug metabolism and ozanimod pharmacokinetics. Previous studies have shown that this duration is sufficient to induce metabolic adaptation to ketogenic diet and establish stable ketosis in mice (Stunault et al. Cell Rep. 2026. doi:10.1016/j.celrep.2026.116945; Tozzi et al. Int J Mol Sci. 2022. doi: 10.3390/ijms232415860). Consistently, in our study, the ketogenic state was confirmed by increased β-hydroxybutyrate levels and altered expression of ketogenesis-related genes. We agree that longer-term studies may provide additional insights into chronic effects and represent an important direction for future research.

**3. Why did you not follow the AIN-93 guidelines as the standard nutritional recommendation for rodents? This recommendation ensures an optimal energy and micronutrient intake for both growth and maintenance phases, thereby eliminating nutritional bias in the experiments. (Line 83)**

The diet selection was based primarily on the need to establish a stable ketogenic state while maintaining comparability between groups. Therefore, commercially available ketogenic and composition-matched control diets from the same manufacturer were selected. This approach minimized differences unrelated to the dietary intervention itself while ensuring effective induction of ketosis, which was confirmed by increased β-hydroxybutyrate levels and changes in ketogenesis-related markers.

**4. Did you use only 3 animals per time point? The ideal minimum would be 5 per group, per time point. (Line 90)**

We agree that the limited number of animals used for the pharmacokinetic assessment represents a limitation of the study and reduces statistical power. The pharmacokinetic experiment was designed as an initial exploratory evaluation to determine whether ketogenic diet may affect ozanimod exposure and whether this interaction warrants further investigation. Therefore, only a limited number of animals was included at this stage. Although the approximately 17% increase in ozanimod exposure did not reach statistical significance, the observed trend suggests a potential effect of KD on ozanimod pharmacokinetics and supports further investigation in larger cohorts and disease-relevant settings.

**5. In which cardiac structure was the puncture performed? This must be described in the text. (Line 97)**

Blood collection was performed by cardiac puncture through both atria (left and right atrium) to obtain sufficient blood volume for subsequent analyses. This information has been added to the Methods section (Page 5, line 122).

**6. Was there any fasting period prior to euthanasia? (Line 96)**

No fasting period was implemented prior to euthanasia. As the primary aim of the study was to characterize the physiological effects of the dietary interventions themselves, introducing a fasting period would have confounded the results by superimposing fasting-induced metabolic changes on diet-induced ones. Mice were therefore maintained on their respective experimental diets *ad libitum* until the time of euthanasia. This information has been added to the Methods section (Page 5, Line 118).

**7. Were the animals fasted for the blood glucose measurement? (Line 104)**

Animals were not fasted prior to blood glucose or ketone measurements. This was a deliberate design decision, as fasting is an independent inducer of ketosis in both humans and mice, and its introduction would have confounded the metabolic readouts by superimposing fasting-driven ketogenesis on the dietary effects under investigation. Measurements were therefore performed under *ad libitum* feeding conditions to capture the metabolic state associated with the dietary intervention. This information has been added to the Methods section (Page 5, line 118).

**8. Similarly, were the animals fasted for a certain period for the lipid profile analysis? This data is highly relevant, given that the study aims to evaluate the influence of the ketogenic diet on the hepatic and pharmacokinetic metabolism of ozanimod. (Line 119)**

As with the blood glucose and ketone measurements, the animals were not fasted prior to lipid profile analysis. The rationale remains consistent with our previous responses: the primary objective was to study the steady-state metabolic effects of the ketogenic diet rather than the effects of fasting. Because fasting alters hepatic lipid metabolism and systemic metabolic state independently of dietary intervention, introducing a fasting period could have confounded interpretation of the observed changes. Therefore, animals remained under *ad libitum* feeding conditions throughout the experiment. Information regarding fasting conditions prior to euthanasia and metabolic measurements has been added to the Methods section (Page 5, line 118).

**9. Please include a detailed statistical analysis in the text. (Line 183)**

The Statistical Analysis section has been expanded to provide a more detailed description of the statistical methods used throughout the study (including the Gut microbiota analysis part). (Page 10, line 219). Details of each statistical test used are provided in the figure legends.

**10. Regarding the total antioxidant capacity results, why was an analysis of hepatic antioxidant enzymes not performed? It would be interesting to add these data, such as SOD, GPx, and GSH. The hepatic redox ratio: SOD + GPx + GSH (non-enzymatic). (Line 195) Note: This could involve applications related to Glutathione (GPx) and mitochondrial dysfunction (SOD).**

We agree that assessment of specific antioxidant pathways, including SOD, GPx, and GSH, would provide a more detailed characterization of hepatic oxidative status. In the present study, FRAP was included as an exploratory marker of overall antioxidant capacity. FRAP did not indicate substantial alterations under the present conditions, and therefore further assessment of specific antioxidant pathways was not pursued. In addition, the amount of biological material obtainable from individual mice was limited considering the broad range of analyses performed, and inclusion of additional oxidative stress markers would have required a substantially larger number of animals.

**11. Regarding the gene expression results, why were they not complemented with a Western Blot (WB)? I believe it would be interesting to verify the protein expression of these genes. (Line 168)**

We agree that protein-level validation may provide additional mechanistic information. However, in the present study, the primary aim was to assess transcriptional changes together with functional outcomes represented by enzyme activity measurements. Therefore, gene expression analysis was complemented by activity assays rather than protein quantification, as enzyme activity was considered the more relevant functional readout for the objectives of this study.

**12. Regarding the results of plasma ozanimod levels, I believe that the very small sample size (n) is a limiting factor for the analysis performed.**

We agree that the limited sample size represents a limitation of the pharmacokinetic analysis and reduces statistical power. This limitation has now been emphasized more clearly in the Discussion section (Line 427) in the manuscript and the pharmacokinetic findings are interpreted cautiously as a non-significant trend rather than a definitive effect.

**Reviewer #2: PONE-D-26-20026 - Ketogenic diet-induced changes in hepatic drug metabolism and ozanimod pharmacokinetics in mice.**

The manuscript investigates whether a ketogenic diet (KD) alters hepatic drug-metabolizing enzymes and the pharmacokinetics of ozanimod, a sphingosine-1-phosphate receptor modulator used in multiple sclerosis. The study combines metabolic characterization, microbiome profiling, hepatic gene expression, CYP activity assays, and pharmacokinetic analyses. The topic is timely and potentially relevant, given the growing interest in ketogenic diets as adjunctive interventions in neurological disorders and the increasing use of ozanimod in clinical practice. The hypothesis that diet-induced metabolic changes may influence drug disposition is biologically plausible and supported by the existing literature, which demonstrates interactions among nutrition, inflammation, gut microbiota, and hepatic cytochrome P450 (CYP) activity. Nevertheless, several important methodological and conceptual limitations substantially weaken the conclusions. In its current form, the study should be regarded as exploratory and hypothesis-generating rather than providing definitive evidence that KD meaningfully alters ozanimod pharmacokinetics.

We sincerely thank Reviewer #2 for the thorough evaluation of our manuscript and for the constructive and insightful comments. We appreciate the reviewer’s recognition of the relevance of our study and the thoughtful suggestions regarding experimental design, interpretation, and methodological rigor, which helped us improve the clarity and balance of the manuscript. Our point-by-point responses are provided below.

**1. Experimental Design: There is disconnect between the breadth of the mechanistic conclusions proposed and the evidence generated.**

a. The study was performed exclusively in healthy young female mice rather than in an experimental model of multiple sclerosis or neuroinflammation. Consequently, the translational relevance to patients receiving ozanimod for autoimmune disease remains uncertain. The metabolic, inflammatory, microbiome, and hepatic responses observed in healthy animals may differ substantially from those occurring in the context of chronic inflammatory disease.

The Reviewer raises an important point regarding the translational relevance of the study design. The use of healthy mice limits direct extrapolation of the findings to patients receiving ozanimod for autoimmune disease. The aim of the present study was not to model multiple sclerosis or neuroinflammation, but to provide an initial mechanistic assessment of whether ketogenic diet may influence hepatic drug metabolism and ozanimod pharmacokinetics under controlled conditions. The use of healthy animals allowed evaluation of diet-associated metabolic and pharmacokinetic changes under controlled conditions and without additional effects introduced by disease-related processes. We acknowledge that the observed metabolic, inflammatory, microbiome, and hepatic changes should be interpreted more cautiously as exploratory associations identifying potential pathways through which ketogenic diet could influence drug disposition. This limitation has now been acknowledged in the revised Discussion (Page 20, line 450).

b. In addition, KD used in this study provided 90% of calories from fat, representing an extreme dietary intervention that may not adequately reflect ketogenic regimens typically used in clinical practice. Therefore, caution is warranted when extrapolating these findings to human patients.

We acknowledge that the ketogenic diet used in this study, providing 90% of calories from fat, represents an experimental dietary model and does not directly reflect ketogenic regimens typically used in clinical practice. The selected dietary composition was intended to reliably induce and maintain a stable ketogenic state and to enable controlled evaluation of diet-associated effects on hepatic metabolism and ozanimod pharmacokinetics. In murine models, higher dietary fat content is commonly required to achieve stable nutritional ketosis due to species-specific metabolic differences (Shou et al., Nutrients. 2025 Oct 12;17(20):3203.). Moreover, therapeutic ketogenic diets used in neurological disorders are generally more restrictive than ketogenic diets used for metabolic conditions such as diabetes or obesity, as well as lifestyle ketogenic diets.

**2. Female Reproductive Status: No information is provided regarding estrous-cycle monitoring, synchronization, or incorporation of reproductive status into the analyses.**

a. The estrous cycle is a well-established source of biological variability affecting hepatic CYP expression, inflammatory responses, gut microbiota composition, and drug pharmacokinetics. Failure to monitor or control for the estrous stage introduces a potentially important confounding variable.

We address these points collectively in the consolidated response below.

b. The manuscript does not indicate whether vaginal cytology was performed, whether animals were sacrificed at comparable estrous stages, or whether sample collection was balanced across cycle phases.

We thank the Reviewer for raising this important point. We agree that reproductive status and hormonal fluctuations may contribute to variability in hepatic CYP expression, inflammatory responses, gut microbiota composition, and pharmacokinetic parameters. However, the aim of the present study was not to evaluate sex- or estrous cycle–dependent differences, but rather to provide an initial assessment of the interaction between ketogenic diet and ozanimod metabolism under standardized experimental conditions. Accordingly, vaginal cytology, estrous-cycle monitoring, synchronization, and stratification were not included in the study design. Future studies may specifically address the contribution of reproductive status and evaluate possible sex-specific responses in greater detail.

**3. Experimental Rigor: The manuscript lacks critical information regarding measures designed to reduce experimental bias.**

a. No randomization procedures are described.

Animals were assigned to experimental groups before the start of the study using weight-based randomization to achieve comparable baseline body weight distribution between groups. This information has been added to the Methods section (Page 5, line 105).

b. No indication that investigators were blinded during sample collection, biochemical analyses, microbiome analysis, gene-expression measurements, enzyme activity assays, pharmacokinetic determinations, or data interpretation.

Formal blinding procedures were not incorporated into the study design and this information has now been clarified in the revised manuscript (Page 5, line 106).

**4. Statistical Power: The study lacks a priori sample size calculation or a statistical power analysis.**

a. This concern is particularly relevant for the pharmacokinetic component. The pharmacokinetic study was performed using only three animals per time point per group. Such a small sample size severely limits statistical power and may explain why the reported increase in ozanimod exposure did not reach statistical significance, despite being emphasized throughout the manuscript.

We address these points collectively in the consolidated response below.

b. Because the study's principal conclusion concerns the influence of KD on ozanimod pharmacokinetics, the limited power of this experiment is a significant weakness.

We acknowledge that the limited sample size of the pharmacokinetic experiment reduces statistical power and limits the strength of conclusions regarding ozanimod exposure. One of the aims of this study was to evaluate whether ketogenic diet-induced changes in hepatic metabolism may also affect ozanimod pharmacokinetics. Although higher ozanimod exposure was observed in KD-fed mice, this difference did not reach statistical significance and should therefore be interpreted cautiously. We have revised the manuscript to further moderate the interpretation of these findings (Discussion, Pages 19, line 427)

**5. Extensive Use of Pooled Samples: The most important methodological limitation of the manuscript is the extensive use of pooled samples.**

a. Measurements of plasma cholesterol, HDL, triglycerides, leptin, IL-6, IL-1β, TNF-α, and several CYP activities were performed using pooled samples from each experimental group, eliminating biological replication and preventing valid statistical inference regarding treatment effects.

We address these points collectively in the consolidated response below.

b. Technical triplicates performed on pooled samples do not constitute biological replicates and cannot be used to estimate inter-animal variability. Consequently, conclusions regarding inflammatory status, lipid metabolism, leptin regulation, and certain CYP activities are substantially weaker than implied by the text.

We address these points collectively in the consolidated response below.

c. Particularly concerning is the interpretation of pooled cytokine measurements as evidence of a KD-induced inflammatory phenotype. Because only a single pooled sample appears to have been analyzed per group, these findings should be interpreted as descriptive observations rather than statistically validated biological effects.

We agree that measurements performed using pooled samples should be interpreted cautiously and do not allow assessment of inter-individual variability. These parameters were included primarily to provide contextual information regarding the metabolic and physiological adaptation induced by ketogenic diet rather than to establish definitive conclusions regarding inflammatory or lipid-related phenotypes. The principal objective of the study was to evaluate whether ketogenic diet-associated metabolic changes may also be accompanied by alterations in hepatic drug metabolism and ozanimod pharmacokinetics. Therefore, parameters directly related to this objective, including key CYP enzymes involved in ozanimod metabolism and pharmacokinetic measurements, were preferentially assessed using individual biological samples whenever feasible. We have revised the manuscript to better distinguish descriptive observations from statistically supported findings. Total cholesterol and triglycerides were measured using individual (non-pooled) plasma samples (n = 8 per group), and the Figure 1 legend has been revised accordingly. Also, Figure 4 has been split into 4A and 4B for clarity.

**6. Statistical Analysis: Although the authors state that normality was assessed using the Shapiro-Wilk test, important aspects of statistical analysis remain insufficiently described.**

a. The Shapiro-Wilk test fails when analyzing a too-small sample.

We have revised the statistical analysis throughout the manuscript to use non-parametric tests, given the relatively small sample size (n = 8 per group), which limits reliable assessment of normality. Specifically, the Mann-Whitney U test was used for pairwise group comparisons, and the relevant sections of the manuscript have been revised accordingly (Page 10, line 219).

b. There is no indication that homogeneity of variance was assessed before applying parametric tests. Furthermore, several datasets appear to involve repeated measurements over time, yet the statistical methods do not account for repeated observations. For example, glucose and β-hydroxybutyrate concentrations were measured repeatedly throughout the intervention period. Analysis of such data using multiple independent t-tests or Mann-Whitney tests is not optimal because these methods ignore within-subject correlations. Mixed-effects models or repeated-measures ANOVA would be more appropriate.

We fully agree that the repeated nature of blood β-hydroxybutyrate and glycaemia measurements requires a method that accounts for within-subject correlation. We have therefore revised the analysis of these longitudinal data using a two-way mixed-effects model with REML estimation and Geisser-Greenhouse correction, with diet as a between-subjects factor and time as a within-subjects factor. Importantly, blood sampling was performed using a rotational design across animals to minimize stress from frequent sampling, meaning not all animals were measured at every timepoint. The REML-based mixed-effects model is particularly appropriate for this design, as it correctly handles the resulting incomplete repeated-measures structure without assuming complete data across all subjects and timepoints. The Methods section (Page 10, line 223) and figure legends have been updated accordingly.

c. Similarly, pharmacokinetic data are inherently longitudinal and should be analyzed using established pharmacokinetic approaches rather than isolated comparisons at individual time points.

However, the pharmacokinetic experiment employed a destructive (sparse) sampling design, in which three different animals were sacrificed at each time point (0, 2, 4, 6, 8, and 24 h). As individual pharmacokinetic profiles cannot be constructed from such data, the dataset is not longitudinal in the repeated-measures sense, and standard non-compartmental or longitudinal modeling approaches are therefore not applicable. Instead, Bailer’s method was used, as it was specifically developed for statistical comparison of AUC estimates derived from destructive sampling designs. To avoid any ambiguity, we have clarified the sampling procedure in the Methods section (Animals - page 5, line 113; and Statistical Analysis – page 10, line 228) and in the legend to Fig. 6.

**7. Pharmacokinetic Interpretation: The central conclusion of the manuscript is that KD alters the pharmacokinetics of ozanimod. However, the experimental evidence supporting this claim is relatively weak.**

a. The reported increase in ozanimod AUC did not achieve statistical significance. Therefore, the data do not demonstrate a definitive pharmacokinetic effect. At most, they suggest a trend that warrants further investigation.

We address these points collectively in the consolidated response below.

b. More importantly, only the parent compound was quantified. Ozanimod undergoes extensive metabolism, and several active metabolites contribute substantially to its pharmacological activity. Without measurement of these metabolites, it is impossible to determine the functional consequences of the observed CYP alterations.

We address these points collectively in the consolidated response below.

c. Indeed, increased exposure to the parent drug could theoretically coexist with reduced formation of active metabolites and unchanged or even reduced pharmacological efficacy. Therefore, statements regarding potential effects on therapeutic activity are not directly supported by the data presented.

We appreciate this comment and agree that the pharmacokinetic findings should be interpreted cautiously. Although higher ozanimod exposure was observed in KD-fed mice, this difference did not reach statistical significance and therefore should be interpreted as a trend rather than a definitive pharmacokinetic effect.

We also acknowledge that measurement of the parent compound alone does not provide a complete assessment of ozanimod disposition or allow conclusions regarding overall pharmacological activity. Because ozanimod undergoes extensive metabolism and active metabolites contribute to its therapeutic effect, the functional consequences of the observed changes in parent compound exposure cannot be determined from the present study. The manuscript has been revised to further clarify these limitations (Discussion - page 19, line 427).

**8. Mechanistic Conclusions: The manuscript proposes that KD influences ozanimod disposition through interactions involving hepatic inflammation, gut microbiota, and CYP regulation. While this hypothesis is plausible, the data presented are primarily correlational.**

a. No experiments were performed to establish causality between alterations in the microbiome and CYP regulation. No microbial metabolites were measured. No receptor activation studies were conducted. Protein expression analyses are absent. Histological assessment of the liver was not performed.

We address these points collectively in the consolidated response below.

b. Consequently, mechanistic interpretations should be substantially tempered. The data demonstrate associations among dietary intervention, microbiome composition, inflammatory markers, and CYP expression, but they do not establish causal relationships among these variables.

We agree that the present study does not establish causal relationships between ketogenic diet-induced changes in gut microbiota, inflammation, hepatic CYP regulation, and ozanimod disposition. Establishing causality would require specifically designed experiments targeting individual pathways, such as microbial metabolites, receptor activation, or isolated modulation of hepatic enzyme regulation, which would be beyond the scope of the present study. The aim here was to address whether concomitant exposure to ketogenic diet and ozanimod may be associated with changes in pathways relevant to drug metabolism and disposition. Therefore, the observed changes are interpreted as associations and potential contributing mechanisms rather than definitive causal links. The Discussion has been revised to further clarify these limitations (from page 16, line 359).

**9. Absence of Histopathological Evaluation: The authors repeatedly discuss the possibility of hepatic inflammation and altered liver function induced by KD. However, no histological assessment of liver tissue was performed.**

a. Histopathological evaluation could have provided important evidence regarding steatosis, inflammatory infiltration, hepatocellular injury, or fibrosis.

We acknowledge that histopathological evaluation could provide additional information regarding structural liver changes associated with ketogenic diet. No clear indications suggesting major hepatic alterations were observed based on the evaluated parameters and histological assessment was therefore not included in the study design. Since the primary objective was not to assess liver pathology but to investigate metabolic and pharmacokinetic changes associated with ketogenic diet, histology was considered complementary rather than essential for interpretation of the present findings.

**Minor Comments**

Introduction: The clinical evidence supporting KDs in multiple sclerosis remains relatively limited. The introduction would benefit from a more balanced discussion of the current level of evidence supporting ketogenic dietary interventions in MS patients.

**Methodological Reporting: Additional methodological details would improve reproducibility.**

a. The microbiome section should provide information regarding sequencing depth, read quality filtering, normalization procedures, and criteria used for taxonomic assignments.

Because the DNA extraction, sequencing, and bioinformatics pipeline were outsourced to a commercial provider (TransnetYX and One Codex), we have updated the "Gut microbiota analysis" section of Methods to include the most critical parameter – the targeted sequencing depth (2 million 2x150 bp read pairs per sample) – while citing the provider's official documentation for their proprietary pipeline details.

To answer your specific questions: taxonomic assignment relies on an exact alignment k-mer-based classification (k=31) against a database of >148,000 reference genomes. Automated artifact filtering based on the relative frequency of unique k-mers is used for read quality control. Regarding normalization, the One Codex pipeline calculates species-level relative abundances by estimating the depth and coverage of sequencing across every available reference genome. A direct link to the detailed TransnetYX/One Codex methodology covering these steps has been added to the Methods section (https://docs.onecodex.com/en/articles/6891294-transnetyx-sequencing-and-analysis-methods).

b. For qPCR analyses, information regarding primer efficiencies and validation of the reference gene under ketogenic dietary conditions would strengthen the methodology.

Gene expression analysis was performed using commercially available TaqMan Gene Expression Assays (Thermo Fisher Scientific), whose primer and probe efficiencies are pre-validated and guaranteed by the manufacturer. The respective Assay IDs have been added to Methods section (Table 1).

*Hprt* was selected based on evidence of its expression stability in murine hepatic tissue (Wang et al. Int J Mol Med. 2018, doi: 10.3892/ijmm.2018.3527). Furthermore, Hprt encodes an enzyme of the purine salvage pathway, which is functionally unrelated to fatty acid oxidation or ketone body metabolism.

**Figures and Data Presentation: The distinction between biological replicates and technical replicates should be more prominently stated throughout the manuscript and figure legends.**

a. The exact "n" of animals for each assessment should be informed.

b. Error bars derived from technical replicates of pooled samples may be misleading because they do not represent biological variability. Readers could incorrectly interpret these values as reflecting inter-animal variation.

Additional methodological details have been added where appropriate to improve reproducibility and transparency. We have also revised the figure legends and methodological descriptions to ensure that the exact number of animals included in each analysis, the use of biological and technical replicates, and sample pooling are stated consistently throughout the manuscript. In particular, Figure 4 now clearly distinguishes between non-pooled (Fig. 4A) and pooled (Fig. 4B) measurements.

**Discussion: Some interpretations go beyond what the data can support.**

a. Statements suggesting potential effects on ozanimod efficacy should be moderated because neither active metabolites nor pharmacodynamic outcomes were measured. Similarly, mechanistic links among microbiota, inflammation, and CYP regulation should be presented more cautiously as hypotheses rather than demonstrated pathways.

We thank the Reviewer for this comment. As discussed above, the Discussion has been revised to present the findings more cautiously and to avoid interpretations beyond the scope of the data (Pages 16-20).
