# Supplementary material for: Ketogenic diet–induced changes in hepatic drug metabolism with potential implications for ozanimod pharmacokinetics in mice
Source: PLoS One. 2026 Sep 8;21(9):e0357797. doi: 10.1371/journal.pone.0357797 (PMC13552735; doi:10.1371/journal.pone.0357797)
Supplement: Supplementary file 2 [file pone.0357797.s003.docx]

We sincerely thank the reviewer for the careful evaluation of our manuscript and for the constructive comments, which helped us improve its clarity and scientific accuracy. In response to the review, we revised the manuscript throughout, including the Title, Abstract, Results, Discussion, and Conclusions, to ensure that the interpretation of our findings more accurately reflects the scope and limitations of the data. In particular, we refined the interpretation of the pharmacokinetic findings, clearly distinguished statistically supported findings from observed trends, and emphasized the limitations of the present study. All revisions are highlighted in the revised manuscript. We believe that these changes have improved the overall accuracy and balance of the manuscript.
