# Supplementary material for: Ketogenic diet–induced changes in hepatic drug metabolism with potential implications for ozanimod pharmacokinetics in mice
Source: PLoS One. 2026 Sep 8;21(9):e0357797. doi: 10.1371/journal.pone.0357797 (PMC13552735; doi:10.1371/journal.pone.0357797)
Supplement: Supplementary file 3 [file pone.0357797.s004.docx]

We sincerely thank the reviewer for the careful re-evaluation of our manuscript and for the constructive comments. We greatly appreciate the positive assessment of the revised manuscript and the suggestions aimed at further improving the precision and balance of the interpretation of our findings.

In response, we carefully reviewed the manuscript once again, with particular attention to the pharmacokinetic findings, measurements obtained from pooled samples, and the proposed mechanistic explanations. We further revised the Abstract and Discussion to consistently present the pharmacokinetic findings as a non-significant trend toward increased ozanimod exposure. We also refined the interpretation of measurements obtained from pooled samples to avoid inferential conclusions and further framed the proposed mechanistic relationships as hypotheses rather than established causal links.

We believe that these revisions have further improved the accuracy of the manuscript. All changes are highlighted in the revised version. We thank the reviewer again for the thoughtful and constructive assessment of our work.
