# Supplementary material for: Ketogenic diet–induced changes in hepatic drug metabolism with potential implications for ozanimod pharmacokinetics in mice
Source: PLoS One. 2026 Sep 8;21(9):e0357797. doi: 10.1371/journal.pone.0357797 (PMC13552735; doi:10.1371/journal.pone.0357797)
Supplement: Peer review history

# Peer review: Decision Letter 0

**DOI:** 10.1371/journal.pone.0357797.r001

**Authors:** Marcia Aguila

2 Jun 2026

Dear Dr. Jourová,

Thank you for submitting your manuscript to PLOS ONE. After careful consideration, we feel that it has merit but does not fully meet PLOS ONE’s publication criteria as it currently stands. Therefore, we invite you to submit a revised version of the manuscript that addresses the points raised during the review process.

Two independent reviewers have evaluated the manuscript and recommended publication after some amendments they have indicated. The responses should be addressed in a revised version. I will be glad if you consider the suggestions raised by the two reviewers and address the reviewers' suggestions as a revised version. Furthermore, the revised version will need to be seen by the reviewers, and a final decision will be based on their assessment.

Please submit your revised manuscript by Jul 17 2026 11:59PM. If you will need more time than this to complete your revisions, please reply to this message or contact the journal office at plosone@plos.org. When you're ready to submit your revision, log on to https://www.editorialmanager.com/pone/ and select the 'Submissions Needing Revision' folder to locate your manuscript file.

- A letter that responds to each point raised by the academic editor and reviewer(s). You should upload this letter as a separate file labeled 'Response to Reviewers'.
- A marked-up copy of your manuscript that highlights changes made to the original version. You should upload this as a separate file labeled 'Revised Manuscript with Track Changes'.
- An unmarked version of your revised paper without tracked changes. You should upload this as a separate file labeled 'Manuscript'.

If you would like to make changes to your financial disclosure, please include your updated statement in your cover letter. Guidelines for resubmitting your figure files are available below the reviewer comments at the end of this letter.

If applicable, we recommend that you deposit your laboratory protocols in protocols.io to enhance the reproducibility of your results. Protocols.io assigns your protocol its own identifier (DOI) so that it can be cited independently in the future. For instructions see: https://journals.plos.org/plosone/s/submission-guidelines#loc-laboratory-protocols. Additionally, PLOS ONE offers an option for publishing peer-reviewed Lab Protocol articles, which describe protocols hosted on protocols.io. Read more information on sharing protocols at https://plos.org/protocols?utm_medium=editorial-email&utm_source=authorletters&utm_campaign=protocols.

As the corresponding author, your ORCID iD is verified in the submission system and will appear in the published article. PLOS supports the use of ORCID, and we encourage all coauthors to register for an ORCID iD and use it as well. Please encourage your coauthors to verify their ORCID iD within the submission system before final acceptance, as unverified ORCID iDs will not appear in the published article. *Only*  the individual author can complete the verification step; PLOS staff *cannot*  verify ORCID iDs on behalf of authors.

We look forward to receiving your revised manuscript.

Kind regards,

Marcia B. Aguila, Ph.D.

Academic Editor

PLOS One

Journal requirements:

When submitting your revision, we need you to address these additional requirements.

1. Please ensure that your manuscript meets PLOS ONE’s style requirements, including those for file naming. The PLOS ONE style templates can be found at

https://journals.plos.org/plosone/s/file?id=wjVg/PLOSOne_formatting_sample_main_body.pdf and

https://journals.plos.org/plosone/s/file?id=ba62/PLOSOne_formatting_sample_title_authors_affiliations.pdf

2. To comply with PLOS One submissions requirements, in your Methods section, please provide additional information regarding the experiments involving animals and ensure you have included details on (1) methods of anesthesia and/or analgesia, and (2) efforts to alleviate suffering.

3. Thank you for stating the following financial disclosure:

“This study was supported by grants from the Czech Science Foundation (23-05645S), Palacky University students’ projects: IGA_LF_2025_008 and IGA_LF_2025_009, the Czech Academy of Sciences under the Lumina quaeruntur fellowship (LQ200202105), and the Ministry of Education, Youth and Sports of the Czech Republic grant Talking Microbes-understanding microbial interactions within One Health framework (CZ.02.01.01/00/22_008/0004597).”

Please state what role the funders took in the study.  If the funders had no role, please state: "The funders had no role in study design, data collection and analysis, decision to publish, or preparation of the manuscript."

If this statement is not correct you must amend it as needed.

Please include this amended Role of Funder statement in your cover letter; we will change the online submission form on your behalf.

4. PLOS requires an ORCID iD for the corresponding author in Editorial Manager on papers submitted after December 6th, 2016. Please ensure that you have an ORCID iD and that it is validated in Editorial Manager. To do this, go to ‘Update my Information’ (in the upper left-hand corner of the main menu), and click on the Fetch/Validate link next to the ORCID field. This will take you to the ORCID site and allow you to create a new iD or authenticate a pre-existing iD in Editorial Manager.

If the reviewer comments include a recommendation to cite specific previously published works, please review and evaluate these publications to determine whether they are relevant and should be cited. There is no requirement to cite these works unless the editor has indicated otherwise.

[Note: HTML markup is below. Please do not edit.]

Reviewers' comments:

Reviewer’s Responses to Questions

**Comments to the Author**

1. Is the manuscript technically sound, and do the data support the conclusions?

Reviewer #1: Yes

Reviewer #2: Partly

**********

2. Has the statistical analysis been performed appropriately and rigorously?

Reviewer #1: Yes

Reviewer #2: No

**********

3. Have the authors made all data underlying the findings in their manuscript fully available?

The PLOS Data policy

Reviewer #1: Yes

Reviewer #2: Yes

**********

4. Is the manuscript presented in an intelligible fashion and written in standard English?

Reviewer #1: Yes

Reviewer #2: Yes

**********

Reviewer #1: The present study is highly relevant to the scientific community, given the considerable increase in the association of a ketogenic diet with neurological and neurodegenerative diseases. It is essential for studies to evaluate the effect of this dietary pattern on drugs widely used in the treatment of these diseases.

However, there are some points that require revision and a few concerns that should be addressed.

1. Why did you use female subjects, given that they can influence hormone levels?

2. Why was the experiment conducted over only 4 weeks? According to some studies, the ideal approach for analyzing the progression of neurodegenerative diseases is long-term (chronic). (Line 80)

3. Why did you not follow the AIN-93 guidelines as the standard nutritional recommendation for rodents? This recommendation ensures an optimal energy and micronutrient intake for both growth and maintenance phases, thereby eliminating nutritional bias in the experiments. (Line 83)

4. Did you use only 3 animals per time point? The ideal minimum would be 5 per group, per time point. (Line 90)

5. In which cardiac structure was the puncture performed? This must be described in the text. (Line 97)

6. Was there any fasting period prior to euthanasia? (Line 96)

7. Were the animals fasted for the blood glucose measurement? (Line 104)

8. Similarly, were the animals fasted for a certain period for the lipid profile analysis? This data is highly relevant, given that the study aims to evaluate the influence of the ketogenic diet on the hepatic and pharmacokinetic metabolism of ozanimod. (Line 119)

9. Please include a detailed statistical analysis in the text. (Line 183)

10. Regarding the total antioxidant capacity results, why was an analysis of hepatic antioxidant enzymes not performed? It would be interesting to add these data, such as SOD, GPx, and GSH. The hepatic redox ratio: SOD + GPx + GSH (non-enzymatic). (Line 195) Note: This could involve applications related to Glutathione (GPx) and mitochondrial dysfunction (SOD).

11. Regarding the gene expression results, why were they not complemented with a Western Blot (WB)? I believe it would be interesting to verify the protein expression of these genes. (Line 168)

12. Regarding the results of plasma ozanimod levels, I believe that the very small sample size (n) is a limiting factor for the analysis performed.

Reviewer #2: PONE-D-26-20026 - Ketogenic diet-induced changes in hepatic drug metabolism and ozanimod pharmacokinetics in mice.

The manuscript investigates whether a ketogenic diet (KD) alters hepatic drug-metabolizing enzymes and the pharmacokinetics of ozanimod, a sphingosine-1-phosphate receptor modulator used in multiple sclerosis. The study combines metabolic characterization, microbiome profiling, hepatic gene expression, CYP activity assays, and pharmacokinetic analyses. The topic is timely and potentially relevant, given the growing interest in ketogenic diets as adjunctive interventions in neurological disorders and the increasing use of ozanimod in clinical practice. The hypothesis that diet-induced metabolic changes may influence drug disposition is biologically plausible and supported by the existing literature, which demonstrates interactions among nutrition, inflammation, gut microbiota, and hepatic cytochrome P450 (CYP) activity. Nevertheless, several important methodological and conceptual limitations substantially weaken the conclusions. In its current form, the study should be regarded as exploratory and hypothesis-generating rather than providing definitive evidence that KD meaningfully alters ozanimod pharmacokinetics.

1. Experimental Design: There is a disconnect between the breadth of the mechanistic conclusions proposed and the evidence generated.

a. The study was performed exclusively in healthy young female mice rather than in an experimental model of multiple sclerosis or neuroinflammation. Consequently, the translational relevance to patients receiving ozanimod for autoimmune disease remains uncertain. The metabolic, inflammatory, microbiome, and hepatic responses observed in healthy animals may differ substantially from those occurring in the context of chronic inflammatory disease.

b. In addition, KD used in this study provided 90% of calories from fat, representing an extreme dietary intervention that may not adequately reflect ketogenic regimens typically used in clinical practice. Therefore, caution is warranted when extrapolating these findings to human patients.

2. Female Reproductive Status: Mo information is provided regarding estrous-cycle monitoring, synchronization, or incorporation of reproductive status into the analyses.

a. The estrous cycle is a well-established source of biological variability affecting hepatic CYP expression, inflammatory responses, gut microbiota composition, and drug pharmacokinetics. Failure to monitor or control for the estrous stage introduces a potentially important confounding variable.

b. The manuscript does not indicate whether vaginal cytology was performed, whether animals were sacrificed at comparable estrous stages, or whether sample collection was balanced across cycle phases.

3. Experimental Rigor: The manuscript lacks critical information regarding measures designed to reduce experimental bias.

a. No randomization procedures are described.

b. No indication that investigators were blinded during sample collection, biochemical analyses, microbiome analysis, gene-expression measurements, enzyme activity assays, pharmacokinetic determinations, or data interpretation.

4. Statistical Power: The study lacks a priori sample size calculation or a statistical power analysis.

a. This concern is particularly relevant for the pharmacokinetic component. The pharmacokinetic study was performed using only three animals per time point per group. Such a small sample size severely limits statistical power and may explain why the reported increase in ozanimod exposure did not reach statistical significance, despite being emphasized throughout the manuscript.

b. Because the study’s principal conclusion concerns the influence of KD on ozanimod pharmacokinetics, the limited power of this experiment is a significant weakness.

5. Extensive Use of Pooled Samples: The most important methodological limitation of the manuscript is the extensive use of pooled samples.

a. Measurements of plasma cholesterol, HDL, triglycerides, leptin, IL-6, IL-1β, TNF-α, and several CYP activities were performed using pooled samples from each experimental group, eliminating biological replication and preventing valid statistical inference regarding treatment effects.

b. Technical triplicates performed on pooled samples do not constitute biological replicates and cannot be used to estimate inter-animal variability. Consequently, conclusions regarding inflammatory status, lipid metabolism, leptin regulation, and certain CYP activities are substantially weaker than implied by the text.

c. Particularly concerning is the interpretation of pooled cytokine measurements as evidence of a KD-induced inflammatory phenotype. Because only a single pooled sample appears to have been analyzed per group, these findings should be interpreted as descriptive observations rather than statistically validated biological effects.

6. Statistical Analysis: Although the authors state that normality was assessed using the Shapiro-Wilk test, important aspects of statistical analysis remain insufficiently described.

a. The Shapiro-Wilk test fails when analyzing a too-small sample.

b. There is no indication that homogeneity of variance was assessed before applying parametric tests. Furthermore, several datasets appear to involve repeated measurements over time, yet the statistical methods do not account for repeated observations. For example, glucose and β-hydroxybutyrate concentrations were measured repeatedly throughout the intervention period. Analysis of such data using multiple independent t-tests or Mann-Whitney tests is not optimal because these methods ignore within-subject correlations. Mixed-effects models or repeated-measures ANOVA would be more appropriate.

c. Similarly, pharmacokinetic data are inherently longitudinal and should be analyzed using established pharmacokinetic approaches rather than isolated comparisons at individual time points.

7. Pharmacokinetic Interpretation: The central conclusion of the manuscript is that KD alters the pharmacokinetics of ozanimod. However, the experimental evidence supporting this claim is relatively weak.

a. The reported increase in ozanimod AUC did not achieve statistical significance. Therefore, the data do not demonstrate a definitive pharmacokinetic effect. At most, they suggest a trend that warrants further investigation.

b. More importantly, only the parent compound was quantified. Ozanimod undergoes extensive metabolism, and several active metabolites contribute substantially to its pharmacological activity. Without measurement of these metabolites, it is impossible to determine the functional consequences of the observed CYP alterations.

c. Indeed, increased exposure to the parent drug could theoretically coexist with reduced formation of active metabolites and unchanged or even reduced pharmacological efficacy. Therefore, statements regarding potential effects on therapeutic activity are not directly supported by the data presented.

8. Mechanistic Conclusions: The manuscript proposes that KD influences ozanimod disposition through interactions involving hepatic inflammation, gut microbiota, and CYP regulation. While this hypothesis is plausible, the data presented are primarily correlational.

a. No experiments were performed to establish causality between alterations in the microbiome and CYP regulation. No microbial metabolites were measured. No receptor activation studies were conducted. Protein expression analyses are absent. Histological assessment of the liver was not performed.

b. Consequently, mechanistic interpretations should be substantially tempered. The data demonstrate associations among dietary intervention, microbiome composition, inflammatory markers, and CYP expression, but they do not establish causal relationships among these variables.

9. Absence of Histopathological Evaluation: The authors repeatedly discuss the possibility of hepatic inflammation and altered liver function induced by KD. However, no histological assessment of liver tissue was performed.

a. Histopathological evaluation could have provided important evidence regarding steatosis, inflammatory infiltration, hepatocellular injury, or fibrosis.

Minor Comments

Introduction: The clinical evidence supporting KDs in multiple sclerosis remains relatively limited. The introduction would benefit from a more balanced discussion of the current level of evidence supporting ketogenic dietary interventions in MS patients.

Methodological Reporting: Additional methodological details would improve reproducibility.

a. The microbiome section should provide information regarding sequencing depth, read quality filtering, normalization procedures, and criteria used for taxonomic assignments.

b. For qPCR analyses, information regarding primer efficiencies and validation of the reference gene under ketogenic dietary conditions would strengthen the methodology.

Figures and Data Presentation: The distinction between biological replicates and technical replicates should be more prominently stated throughout the manuscript and figure legends.

a. The exact "n" of animals for each assessment should be informed.

b. Error bars derived from technical replicates of pooled samples may be misleading because they do not represent biological variability. Readers could incorrectly interpret these values as reflecting inter-animal variation.

Discussion: Some interpretations go beyond what the data can support.

a. Statements suggesting potential effects on ozanimod efficacy should be moderated because neither active metabolites nor pharmacodynamic outcomes were measured. Similarly, mechanistic links among microbiota, inflammation, and CYP regulation should be presented more cautiously as hypotheses rather than demonstrated pathways.

**********

what does this mean?). If published, this will include your full peer review and any attached files.

If you choose “no”, your identity will remain anonymous but your review may still be made public.

**Do you want your identity to be public for this peer review?**  For information about this choice, including consent withdrawal, please see our Privacy Policy

Reviewer #1: No

Reviewer #2: No

**********

[NOTE: If reviewer comments were submitted as an attachment file, they will be attached to this email and accessible via the submission site. Please log into your account, locate the manuscript record, and check for the action link "View Attachments". If this link does not appear, there are no attachment files.]

To ensure your figures meet our technical requirements, please review our figure guidelines: https://journals.plos.org/plosone/s/figures

You may also use PLOS’s free figure tool, NAAS, to help you prepare publication quality figures: https://journals.plos.org/plosone/s/figures#loc-tools-for-figure-preparation.

NAAS will assess whether your figures meet our technical requirements by comparing each figure against our figure specifications.

# Author comment: Author response to Decision Letter 1

**DOI:** 10.1371/journal.pone.0357797.r002

11 Jul 2026

Reviewer #1: The present study is highly relevant to the scientific community, given the considerable increase in the association of a ketogenic diet with neurological and neurodegenerative diseases. It is essential for studies to evaluate the effect of this dietary pattern on drugs widely used in the treatment of these diseases.

However, there are some points that require revision and a few concerns that should be addressed.

We sincerely thank Reviewer #1 for the careful evaluation of our manuscript and for the constructive comments and suggestions. We appreciate the positive assessment of the relevance of our study and believe that the reviewer’s comments helped improve the clarity and quality of the manuscript. Our point-by-point responses are provided below.

1. Why did you use female subjects, given that they can influence hormone levels?

Female mice were selected intentionally to increase the translational relevance of the study, as multiple sclerosis occurs more frequently in women (Compston and Coles. Lancet. 2008 doi:10.1016/S0140-6736(08)61620-7) and ozanimod is also administered to female patients. We acknowledge that hormonal fluctuations may influence hepatic metabolism, inflammatory responses, and pharmacokinetics. Future studies may further address the potential contribution of reproductive status and evaluate possible sex-specific responses.

2. Why was the experiment conducted over only 4 weeks? According to some studies, the ideal approach for analyzing the progression of neurodegenerative diseases is long-term (chronic). (Line 80)

The duration of 4 weeks was selected intentionally because the aim of this study was not to model the long-term progression of neurodegenerative disease, but to perform an initial assessment of whether ketogenic diet may influence hepatic drug metabolism and ozanimod pharmacokinetics. Previous studies have shown that this duration is sufficient to induce metabolic adaptation to ketogenic diet and establish stable ketosis in mice (Stunault et al. Cell Rep. 2026. doi:10.1016/j.celrep.2026.116945; Tozzi et al. Int J Mol Sci. 2022. doi: 10.3390/ijms232415860). Consistently, in our study, the ketogenic state was confirmed by increased β-hydroxybutyrate levels and altered expression of ketogenesis-related genes. We agree that longer-term studies may provide additional insights into chronic effects and represent an important direction for future research.

3. Why did you not follow the AIN-93 guidelines as the standard nutritional recommendation for rodents? This recommendation ensures an optimal energy and micronutrient intake for both growth and maintenance phases, thereby eliminating nutritional bias in the experiments. (Line 83)

The diet selection was based primarily on the need to establish a stable ketogenic state while maintaining comparability between groups. Therefore, commercially available ketogenic and composition-matched control diets from the same manufacturer were selected. This approach minimized differences unrelated to the dietary intervention itself while ensuring effective induction of ketosis, which was confirmed by increased β-hydroxybutyrate levels and changes in ketogenesis-related markers.

4. Did you use only 3 animals per time point? The ideal minimum would be 5 per group, per time point. (Line 90)

We agree that the limited number of animals used for the pharmacokinetic assessment represents a limitation of the study and reduces statistical power. The pharmacokinetic experiment was designed as an initial exploratory evaluation to determine whether ketogenic diet may affect ozanimod exposure and whether this interaction warrants further investigation. Therefore, only a limited number of animals was included at this stage. Although the approximately 17% increase in ozanimod exposure did not reach statistical significance, the observed trend suggests a potential effect of KD on ozanimod pharmacokinetics and supports further investigation in larger cohorts and disease-relevant settings.

5. In which cardiac structure was the puncture performed? This must be described in the text. (Line 97)

Blood collection was performed by cardiac puncture through both atria (left and right atrium) to obtain sufficient blood volume for subsequent analyses. This information has been added to the Methods section (Page 5, line 122).

6. Was there any fasting period prior to euthanasia? (Line 96)

No fasting period was implemented prior to euthanasia. As the primary aim of the study was to characterize the physiological effects of the dietary interventions themselves, introducing a fasting period would have confounded the results by superimposing fasting-induced metabolic changes on diet-induced ones. Mice were therefore maintained on their respective experimental diets ad libitum until the time of euthanasia. This information has been added to the Methods section (Page 5, Line 118).

7. Were the animals fasted for the blood glucose measurement? (Line 104)

Animals were not fasted prior to blood glucose or ketone measurements. This was a deliberate design decision, as fasting is an independent inducer of ketosis in both humans and mice, and its introduction would have confounded the metabolic readouts by superimposing fasting-driven ketogenesis on the dietary effects under investigation. Measurements were therefore performed under ad libitum feeding conditions to capture the metabolic state associated with the dietary intervention. This information has been added to the Methods section (Page 5, line 118).

8. Similarly, were the animals fasted for a certain period for the lipid profile analysis? This data is highly relevant, given that the study aims to evaluate the influence of the ketogenic diet on the hepatic and pharmacokinetic metabolism of ozanimod. (Line 119)

As with the blood glucose and ketone measurements, the animals were not fasted prior to lipid profile analysis. The rationale remains consistent with our previous responses: the primary objective was to study the steady-state metabolic effects of the ketogenic diet rather than the effects of fasting. Because fasting alters hepatic lipid metabolism and systemic metabolic state independently of dietary intervention, introducing a fasting period could have confounded interpretation of the observed changes. Therefore, animals remained under ad libitum feeding conditions throughout the experiment. Information regarding fasting conditions prior to euthanasia and metabolic measurements has been added to the Methods section (Page 5, line 118).

9. Please include a detailed statistical analysis in the text. (Line 183)

The Statistical Analysis section has been expanded to provide a more detailed description of the statistical methods used throughout the study (including the Gut microbiota analysis part). (Page 10, line 219). Details of each statistical test used are provided in the figure legends.

10. Regarding the total antioxidant capacity results, why was an analysis of hepatic antioxidant enzymes not performed? It would be interesting to add these data, such as SOD, GPx, and GSH. The hepatic redox ratio: SOD + GPx + GSH (non-enzymatic). (Line 195) Note: This could involve applications related to Glutathione (GPx) and mitochondrial dysfunction (SOD).

We agree that assessment of specific antioxidant pathways, including SOD, GPx, and GSH, would provide a more detailed characterization of hepatic oxidative status. In the present study, FRAP was included as an exploratory marker of overall antioxidant capacity. FRAP did not indicate substantial alterations under the present conditions, and therefore further assessment of specific antioxidant pathways was not pursued. In addition, the amount of biological material obtainable from individual mice was limited considering the broad range of analyses performed, and inclusion of additional oxidative stress markers would have required a substantially larger number of animals.

11. Regarding the gene expression results, why were they not complemented with a Western Blot (WB)? I believe it would be interesting to verify the protein expression of these genes. (Line 168)

We agree that protein-level validation may provide additional mechanistic information. However, in the present study, the primary aim was to assess transcriptional changes together with functional outcomes represented by enzyme activity measurements. Therefore, gene expression analysis was complemented by activity assays rather than protein quantification, as enzyme activity was considered the more relevant functional readout for the objectives of this study.

12. Regarding the results of plasma ozanimod levels, I believe that the very small sample size (n) is a limiting factor for the analysis performed.

We agree that the limited sample size represents a limitation of the pharmacokinetic analysis and reduces statistical power. This limitation has now been emphasized more clearly in the Discussion section (Line 427) in the manuscript and the pharmacokinetic findings are interpreted cautiously as a non-significant trend rather than a definitive effect.

Reviewer #2: PONE-D-26-20026 - Ketogenic diet-induced changes in hepatic drug metabolism and ozanimod pharmacokinetics in mice.

The manuscript investigates whether a ketogenic diet (KD) alters hepatic drug-metabolizing enzymes and the pharmacokinetics of ozanimod, a sphingosine-1-phosphate receptor modulator used in multiple sclerosis. The study combines metabolic characterization, microbiome profiling, hepatic gene expression, CYP activity assays, and pharmacokinetic analyses. The topic is timely and potentially relevant, given the growing interest in ketogenic diets as adjunctive interventions in neurological disorders and the increasing use of ozanimod in clinical practice. The hypothesis that diet-induced metabolic changes may influence drug disposition is biologically plausible and supported by the existing literature, which demonstrates interactions among nutrition, inflammation, gut microbiota, and hepatic cytochrome P450 (CYP) activity. Nevertheless, several important methodological and conceptual limitations substantially weaken the conclusions. In its current form, the study should be regarded as exploratory and hypothesis-generating rather than providing definitive evidence that KD meaningfully alters ozanimod pharmacokinetics.

We sincerely thank Reviewer #2 for the thorough evaluation of our manuscript and for the constructive and insightful comments. We appreciate the reviewer’s recognition of the relevance of our study and the thoughtful suggestions regarding experimental design, interpretation, and methodological rigor, which helped us improve the clarity and balance of the manuscript. Our point-by-point responses are provided below.

1. Experimental Design: There is disconnect between the breadth of the mechanistic conclusions proposed and the evidence generated.

a. The study was performed exclusively in healthy young female mice rather than in an experimental model of multiple sclerosis or neuroinflammation. Consequently, the translational relevance to patients receiving ozanimod for autoimmune disease remains uncertain. The metabolic, inflammatory, microbiome, and hepatic responses observed in healthy animals may differ substantially from those occurring in the context of chronic inflammatory disease.

The Reviewer raises an important point regarding the translational relevance of the study design. The use of healthy mice limits direct extrapolation of the findings to patients receiving ozanimod for autoimmune disease. The aim of the present study was not to model multiple sclerosis or neuroinflammation, but to provide an initial mechanistic assessment of whether ketogenic diet may influence hepatic drug metabolism and ozanimod pharmacokinetics under controlled conditions. The use of healthy animals allowed evaluation of diet-associated metabolic and pharmacokinetic changes under controlled conditions and without additional effects introduced by disease-related processes. We acknowledge that the observed metabolic, inflammatory, microbiome, and hepatic changes should be interpreted more cautiously as exploratory associations identifying potential pathways through which ketogenic diet could influence drug disposition. This limitation has now been acknowledged in the revised Discussion (Page 20, line 450).

b. In addition, KD used in this study provided 90% of calories from fat, representing an extreme dietary intervention that may not adequately reflect ketogenic regimens typically used in clinical practice. Therefore, caution is warranted when extrapolating these findings to human patients.

We acknowledge that the ketogenic diet used in this study, providing 90% of calories from fat, represents an experimental dietary model and does not directly reflect ketogenic regimens typically used in clinical practice. The selected dietary composition was intended to reliably induce and maintain a stable ketogenic state and to enable controlled evaluation of diet-associated effects on hepatic metabolism and ozanimod pharmacokinetics. In murine models, higher dietary fat content is commonly required to achieve stable nutritional ketosis due to species-specific metabolic differences (Shou et al., Nutrients. 2025 Oct 12;17(20):3203.). Moreover, therapeutic ketogenic diets used in neurological disorders are generally more restrictive than ketogenic diets used for metabolic conditions such as diabetes or obesity, as well as lifestyle ketogenic diets.

2. Female Reproductive Status: No information is provided regarding estrous-cycle monitoring, synchronization, or incorporation of reproductive status into the analyses.

a. The estrous cycle is a well-established source of biological variability affecting hepatic CYP expression, inflammatory responses, gut microbiota composition, and drug pharmacokinetics. Failure to monitor or control for the estrous stage introduces a potentially important confounding variable.

We address these points collectively in the consolidated response below.

b. The manuscript does not indicate whether vaginal cytology was performed, whether animals were sacrificed at comparable estrous stages, or whether sample collection was balanced across cycle phases.

We thank the Reviewer for raising this important point. We agree that reproductive status and hormonal fluctuations may contribute to variability in hepatic CYP expression, inflammatory responses, gut microbiota composition, and pharmacokinetic parameters. However, the aim of the present study was not to evaluate sex- or estrous cycle–dependent differences, but rather to provide an initial assessment of the interaction between ketogenic diet and ozanimod metabolism under standardized experimental conditions. Accordingly, vaginal cytology, estrous-cycle monitoring, synchronization, and stratification were not included in the study design. Future studies may specifically address the contribution of reproductive status and evaluate possible sex-specific responses in greater detail.

3. Experimental Rigor: The manuscript lacks critical information regarding measures designed to reduce experimental bias.

a. No randomization procedures are described.

Animals were assigned to experimental groups before the start of the study using weight-based randomization to achieve comparable baseline body weight distribution between groups. This information has been added to the Methods section (Page 5, line 105).

b. No indication that investigators were blinded during sample collection, biochemical analyses, microbiome analysis, gene-expression measurements, enzyme activity assays, pharmacokinetic determinations, or data interpretation.

Formal blinding procedures were not incorporated into the study design and this information has now been clarified in the revised manuscript (Page 5, line 106).

4. Statistical Power: The study lacks a priori sample size calculation or a statistical power analysis.

a. This concern is particularly relevant for the pharmacokinetic component. The pharmacokinetic study was performed using only three animals per time point per group. Such a small sample size severely limits statistical power and may explain why the reported increase in ozanimod exposure did not reach

Attachment Submitted filename: Comments for Reviewers.docx

# Peer review: Decision Letter 1

**DOI:** 10.1371/journal.pone.0357797.r003

**Authors:** Marcia Aguila

27 Jul 2026

Dear Dr. Jourová,

plosone@plos.org. When you're ready to submit your revision, log on to https://www.editorialmanager.com/pone/ and select the 'Submissions Needing Revision' folder to locate your manuscript file.

- A letter that responds to each point raised by the academic editor and reviewer(s). You should upload this letter as a separate file labeled 'Response to Reviewers'.
- A marked-up copy of your manuscript that highlights changes made to the original version. You should upload this as a separate file labeled 'Revised Manuscript with Track Changes'.
- An unmarked version of your revised paper without tracked changes. You should upload this as a separate file labeled 'Manuscript'.

If you would like to make changes to your financial disclosure, please include your updated statement in your cover letter. Guidelines for resubmitting your figure files are available below the reviewer comments at the end of this letter.

If applicable, we recommend that you deposit your laboratory protocols in protocols.io to enhance the reproducibility of your results. Protocols.io assigns your protocol its own identifier (DOI) so that it can be cited independently in the future. For instructions see: https://journals.plos.org/plosone/s/submission-guidelines#loc-laboratory-protocols. Additionally, PLOS ONE offers an option for publishing peer-reviewed Lab Protocol articles, which describe protocols hosted on protocols.io. Read more information on sharing protocols at https://plos.org/protocols?utm_medium=editorial-email&utm_source=authorletters&utm_campaign=protocols.

As the corresponding author, your ORCID iD is verified in the submission system and will appear in the published article. PLOS supports the use of ORCID, and we encourage all coauthors to register for an ORCID iD and use it as well. Please encourage your coauthors to verify their ORCID iD within the submission system before final acceptance, as unverified ORCID iDs will not appear in the published article. *Only*  the individual author can complete the verification step; PLOS staff *cannot*  verify ORCID iDs on behalf of authors.

We look forward to receiving your revised manuscript.

Kind regards,

Marcia B. Aguila, Ph.D.

Academic Editor

PLOS One

Journal Requirements:

If the reviewer comments include a recommendation to cite specific previously published works, please review and evaluate these publications to determine whether they are relevant and should be cited. There is no requirement to cite these works unless the editor has indicated otherwise.

Please review your reference list to ensure that it is complete and correct. If you have cited papers that have been retracted, please include the rationale for doing so in the manuscript text, or remove these references and replace them with relevant current references. Any changes to the reference list should be mentioned in the rebuttal letter that accompanies your revised manuscript. If you need to cite a retracted article, indicate the article’s retracted status in the References list and also include a citation and full reference for the retraction notice.

**Additional Editor Comments:**

The authors have satisfactorily addressed the first reviewer’s comments. However, the second reviewer still raises important points that require further attention. We kindly ask you to carefully consider these issues and respond to them in a revised submission. The revised version will be sent back to the second reviewer, who will have the final decision.

[Note: HTML markup is below. Please do not edit.]

Reviewers' comments:

Reviewer’s Responses to Questions

**Comments to the Author**

Reviewer #1: All comments have been addressed

Reviewer #2: (No Response)

**********

2. Is the manuscript technically sound, and do the data support the conclusions?

Reviewer #1: Yes

Reviewer #2: Partly

**********

3. Has the statistical analysis been performed appropriately and rigorously?

Reviewer #1: Yes

Reviewer #2: Yes

**********

4. Have the authors made all data underlying the findings in their manuscript fully available?

The PLOS Data policy

Reviewer #1: Yes

Reviewer #2: Yes

**********

5. Is the manuscript presented in an intelligible fashion and written in standard English?

Reviewer #1: Yes

Reviewer #2: Yes

**********

Reviewer #1: (No Response)

Reviewer #2: The authors have carefully responded to the previous comments and made several meaningful revisions that improve transparency, statistical handling, and the overall balance of the manuscript. In particular, the revised version now more clearly describes the allocation of animals, acknowledges the absence of formal blinding, distinguishes pooled from individual biological samples, applies a mixed-effects model to the repeated metabolic measurements, and clarifies the destructive sampling design used for the pharmacokinetic analysis. The use of Bailer’s method for comparison of AUC estimates derived from sparse terminal sampling is appropriate for this experimental design. The authors have also moderated several mechanistic interpretations and now recognize that the associations among ketogenic diet, microbiota composition, inflammatory markers, hepatic CYP regulation, and ozanimod exposure do not establish causal relationships. These changes substantially improve methodological reporting and make the study’s scope more explicit.

1. Nevertheless, the principal limitations identified in the initial review remain inherent to the experimental design and should continue to frame the interpretation of the findings. The study was conducted exclusively in healthy young female mice receiving an experimental ketogenic diet containing 90% of energy from fat. This model is useful for establishing stable ketosis under controlled conditions, but it does not reproduce the inflammatory and immunological environment in which ozanimod is clinically administered. Direct extrapolation to patients with multiple sclerosis therefore remains limited. In addition, the estrous cycle stage was neither monitored nor balanced across groups. The authors appropriately acknowledge this point, but reproductive status remains a potentially important uncontrolled source of variability for hepatic CYP expression, inflammatory mediators, microbiota composition, and drug disposition. The absence of formal blinding is another limitation that cannot be corrected retrospectively, although its explicit disclosure is welcome.

2. The extensive use of pooled samples also continues to restrict the evidential value of several measurements. Technical replicates obtained from a single pooled sample do not constitute independent biological replicates and cannot be used to estimate inter-animal variability or to support inferential statistical comparisons. The authors have improved the presentation by clearly distinguishing pooled and non-pooled analyses and by reporting cholesterol and triglycerides from individual samples. However, pooled measurements of cytokines, leptin, and selected CYP activities remain descriptive. These data may provide contextual information, but they should not be used to establish a ketogenic diet-induced inflammatory phenotype or a reproducible biological effect. Any remaining statements that imply statistically validated changes based on pooled samples should therefore be revised.

3. The pharmacokinetic component remains the most important limitation of the study. Using only three animals per group at each terminal time point provides limited precision and low statistical power. The approximately 17% increase in ozanimod AUC did not reach statistical significance and should not be described as evidence that the ketogenic diet altered ozanimod pharmacokinetics. At most, the data indicate a preliminary, non-significant trend that may justify further investigation. This distinction is particularly important because only the parent compound was measured. Ozanimod undergoes extensive biotransformation, and active metabolites make a major contribution to its pharmacological activity. Without quantifying these metabolites, the study cannot determine whether altered CYP activity alters total active exposure, metabolite formation, therapeutic efficacy, or toxicity. Increased exposure to the parent drug could occur together with reduced formation of active metabolites, leaving the overall pharmacological effect unchanged or even reduced. Accordingly, the manuscript cannot support conclusions regarding altered therapeutic activity.

4. Although the authors have moderated parts of the Discussion, the conclusions remain somewhat more assertive than warranted by the data. Statements indicating that the ketogenic diet may influence the pharmacokinetic profile of ozanimod, potentially alter its pharmacological activity, or provide the first evidence that the ketogenic diet affects ozanimod disposition should be revised. The results demonstrate changes in selected hepatic CYP expression and activity endpoints and a non-significant tendency toward greater exposure to parent ozanimod. They do not demonstrate a statistically supported pharmacokinetic interaction or any effect on pharmacological activity. The same caution should be applied consistently in the Abstract, Discussion, and concluding paragraph.

5. The mechanistic discussion should also remain explicitly hypothesis-generating. The observed microbiome changes were not experimentally linked to CYP regulation via microbial metabolite measurements, receptor activation studies, microbiota transfer experiments, or pathway-specific interventions. Protein abundance was not assessed, and hepatic histopathology was not performed. Functional CYP activity measurements are valuable and, for the specific purpose of evaluating drug-metabolizing capacity, may be more informative than protein abundance alone. However, the absence of liver histology weakens statements concerning hepatic inflammation, steatosis, injury, or structural adaptation. The argument that there were no indications of substantial liver alterations cannot replace direct morphological evaluation. Any discussion of hepatic pathology should therefore be limited to the biochemical and molecular variables actually measured.

6. The revised manuscript is substantially improved and is now more appropriately positioned as an exploratory, hypothesis-generating investigation of a possible diet–drug interaction. Most methodological concerns have either been addressed through reanalysis and clarification or transparently acknowledged as limitations. No additional large experimental program is required at this stage. However, the concluding language still requires further refinement to accurately reflect the non-significant pharmacokinetic result, the small sample size, the use of pooled samples, and the absence of active-metabolite and pharmacodynamic measurements.

**********

what does this mean?). If published, this will include your full peer review and any attached files.

If you choose “no”, your identity will remain anonymous but your review may still be made public.

**Do you want your identity to be public for this peer review?**  For information about this choice, including consent withdrawal, please see our Privacy Policy

Reviewer #1: No

Reviewer #2: No

**********

[NOTE: If reviewer comments were submitted as an attachment file, they will be attached to this email and accessible via the submission site. Please log into your account, locate the manuscript record, and check for the action link "View Attachments". If this link does not appear, there are no attachment files.]

To ensure your figures meet our technical requirements, please review our figure guidelines: https://journals.plos.org/plosone/s/figures

You may also use PLOS’s free figure tool, NAAS, to help you prepare publication quality figures: https://journals.plos.org/plosone/s/figures#loc-tools-for-figure-preparation.

NAAS will assess whether your figures meet our technical requirements by comparing each figure against our figure specifications.

# Author comment: Author response to Decision Letter 2

**DOI:** 10.1371/journal.pone.0357797.r004

3 Aug 2026

We sincerely thank the reviewer for the careful evaluation of our manuscript and for the constructive comments, which helped us improve its clarity and scientific accuracy. In response to the review, we revised the manuscript throughout, including the Title, Abstract, Results, Discussion, and Conclusions, to ensure that the interpretation of our findings more accurately reflects the scope and limitations of the data. In particular, we refined the interpretation of the pharmacokinetic findings, clearly distinguished statistically supported findings from observed trends, and emphasized the limitations of the present study. All revisions are highlighted in the revised manuscript. We believe that these changes have improved the overall accuracy and balance of the manuscript.

Attachment Submitted filename: Respond to reviewers 2.docx

# Peer review: Decision Letter 2

**DOI:** 10.1371/journal.pone.0357797.r005

**Authors:** Marcia Aguila

19 Aug 2026

Dear Dr. Jourová,

Thank you for submitting your manuscript to PLOS One. After careful consideration, we feel that it has merit but does not fully meet PLOS One’s publication criteria as it currently stands. Therefore, we invite you to submit a revised version of the manuscript that addresses the points raised during the review process.

Please submit your revised manuscript by Oct 03 2026 11:59PM. If you will need more time than this to complete your revisions, please reply to this message or contact the journal office at plosone@plos.org. When you're ready to submit your revision, log on to https://www.editorialmanager.com/pone/ and select the 'Submissions Needing Revision' folder to locate your manuscript file.

- A letter that responds to each point raised by the academic editor and reviewer(s). You should upload this letter as a separate file labeled 'Response to Reviewers'.
- A marked-up copy of your manuscript that highlights changes made to the original version. You should upload this as a separate file labeled 'Revised Manuscript with Track Changes'.
- An unmarked version of your revised paper without tracked changes. You should upload this as a separate file labeled 'Manuscript'.

If you would like to make changes to your financial disclosure, please include your updated statement in your cover letter. Guidelines for resubmitting your figure files are available below the reviewer comments at the end of this letter.

If applicable, we recommend that you deposit your laboratory protocols in protocols.io to enhance the reproducibility of your results. Protocols.io assigns your protocol its own identifier (DOI) so that it can be cited independently in the future. For instructions see: https://journals.plos.org/plosone/s/submission-guidelines#loc-laboratory-protocols. Additionally, PLOS One offers an option for publishing peer-reviewed Lab Protocol articles, which describe protocols hosted on protocols.io. Read more information on sharing protocols at https://plos.org/protocols?utm_medium=editorial-email&utm_source=authorletters&utm_campaign=protocols.

As the corresponding author, your ORCID iD is verified in the submission system and will appear in the published article. PLOS supports the use of ORCID, and we encourage all coauthors to register for an ORCID iD and use it as well. Please encourage your coauthors to verify their ORCID iD within the submission system before final acceptance, as unverified ORCID iDs will not appear in the published article. *Only*  the individual author can complete the verification step; PLOS staff *cannot*  verify ORCID iDs on behalf of authors.

We look forward to receiving your revised manuscript.

Kind regards,

Marcia B. Aguila, Ph.D.

Academic Editor

PLOS One

Journal Requirements:

If the reviewer comments include a recommendation to cite specific previously published works, please review and evaluate these publications to determine whether they are relevant and should be cited. There is no requirement to cite these works unless the editor has indicated otherwise.

Please review your reference list to ensure that it is complete and correct. If you have cited papers that have been retracted, please include the rationale for doing so in the manuscript text, or remove these references and replace them with relevant current references. Any changes to the reference list should be mentioned in the rebuttal letter that accompanies your revised manuscript. If you need to cite a retracted article, indicate the article’s retracted status in the References list and also include a citation and full reference for the retraction notice.

**Additional Editor Comments:**

The authors have adequately addressed the concerns of reviewers, but our second reviewer has yet a question to the authors. This point will need to be addressed in a revised version. I would like to ask you to consider this question raised by the second reviewer.

[Note: HTML markup is below. Please do not edit.]

Reviewers' comments:

Reviewer’s Responses to Questions

**Comments to the Author**

Reviewer #2: (No Response)

**********

2. Is the manuscript technically sound, and do the data support the conclusions?

Reviewer #2: Yes

**********

3. Has the statistical analysis been performed appropriately and rigorously?

Reviewer #2: Yes

**********

4. Have the authors made all data underlying the findings in their manuscript fully available?

The PLOS Data policy

Reviewer #2: Yes

**********

5. Is the manuscript presented in an intelligible fashion and written in standard English?

Reviewer #2: Yes

**********

Reviewer #2: PONE-D-26-20026R2 - Ketogenic diet–induced changes in hepatic drug metabolism with potential implications for ozanimod pharmacokinetics in mice

The manuscript has improved substantially since the previous revision, and the authors have addressed most of the methodological and reporting concerns raised previously. The remaining issues relate primarily to the interpretation of the results rather than to the experimental work itself. These points can be addressed through relatively minor revisions.

Major Comments

1. Interpretation of the pharmacokinetic findings: The authors now correctly state that the approximately 17% increase in ozanimod exposure did not reach statistical significance. However, a few statements still suggest a pharmacokinetic interaction that is not fully supported by the data. Throughout the manuscript, the conclusions should consistently reflect that the study identified only a non-significant trend toward increased exposure to the parent compound. Because active metabolites were not measured, the data do not allow conclusions regarding overall drug exposure, pharmacological activity, or therapeutic efficacy.

2. Interpretation of pooled measurements: The distinction between pooled and individual samples is now much clearer. Nevertheless, measurements obtained from pooled samples (cytokines, leptin, and selected CYP activities) remain descriptive and should not be interpreted as evidence of reproducible biological differences. A final review of the manuscript would help ensure that no inferential language is used when discussing these data.

3. Mechanistic interpretation: The Discussion is considerably more balanced than in the previous version, but the proposed links between the ketogenic diet, gut microbiota, inflammatory pathways, CYP regulation, and ozanimod disposition should remain clearly framed as hypotheses rather than established mechanisms. No mechanistic experiments, active metabolite analyses, or liver histopathology were performed, and the discussion should continue to reflect these limitations.

Minor Comments

The manuscript is well written, and the English is clear throughout. The statistical analyses are appropriate and are now described more transparently. The principal limitations of the study—including the use of healthy female mice, the absence of blinding, the lack of estrous cycle monitoring, and the exploratory nature of the pharmacokinetic analysis—are adequately acknowledged.

**********

what does this mean?). If published, this will include your full peer review and any attached files.

If you choose “no”, your identity will remain anonymous but your review may still be made public.

**Do you want your identity to be public for this peer review?**  For information about this choice, including consent withdrawal, please see our Privacy Policy

Reviewer #2: No

**********

[NOTE: If reviewer comments were submitted as an attachment file, they will be attached to this email and accessible via the submission site. Please log into your account, locate the manuscript record, and check for the action link "View Attachments". If this link does not appear, there are no attachment files.]

To ensure your figures meet our technical requirements, please review our figure guidelines: https://journals.plos.org/plosone/s/figures

You may also use PLOS’s free figure tool, NAAS, to help you prepare publication quality figures: https://journals.plos.org/plosone/s/figures#loc-tools-for-figure-preparation.

NAAS will assess whether your figures meet our technical requirements by comparing each figure against our figure specifications.

# Author comment: Author response to Decision Letter 3

**DOI:** 10.1371/journal.pone.0357797.r006

20 Aug 2026

We sincerely thank the reviewer for the careful re-evaluation of our manuscript and for the constructive comments. We greatly appreciate the positive assessment of the revised manuscript and the suggestions aimed at further improving the precision and balance of the interpretation of our findings.

In response, we carefully reviewed the manuscript once again, with particular attention to the pharmacokinetic findings, measurements obtained from pooled samples, and the proposed mechanistic explanations. We further revised the Abstract and Discussion to consistently present the pharmacokinetic findings as a non-significant trend toward increased ozanimod exposure. We also refined the interpretation of measurements obtained from pooled samples to avoid inferential conclusions and further framed the proposed mechanistic relationships as hypotheses rather than established causal links.

We believe that these revisions have further improved the accuracy of the manuscript. All changes are highlighted in the revised version. We thank the reviewer again for the thoughtful and constructive assessment of our work.

Attachment Submitted filename: Response to Reviewer 3.docx

# Peer review: Decision Letter 3

**DOI:** 10.1371/journal.pone.0357797.r007

**Authors:** Marcia Aguila

23 Aug 2026

Ketogenic diet–induced changes in hepatic drug metabolism with potential implications for ozanimod pharmacokinetics in mice

PONE-D-26-20026R3

Dear Dr. Jourová,

We’re pleased to inform you that your manuscript has been judged scientifically suitable for publication and will be formally accepted for publication once it meets all outstanding technical requirements.

Within one week, you’ll receive an e-mail detailing the required amendments. When these have been addressed, you’ll receive a formal acceptance letter and your manuscript will be scheduled for publication.

An invoice will be generated when your article is formally accepted. Please note, if your institution has a publishing partnership with PLOS and your article meets the relevant criteria, all or part of your publication costs will be covered. Please make sure your user information is up-to-date by logging into Editorial Manager at Editorial Manager® and clicking the ‘Update My Information' link at the top of the page. For questions related to billing, please contact billing support.

If your institution or institutions have a press office, please notify them about your upcoming paper to help maximize its impact. If they’ll be preparing press materials, please inform our press team as soon as possible -- no later than 48 hours after receiving the formal acceptance. Your manuscript will remain under strict press embargo until 2 pm Eastern Time on the date of publication. For more information, please contact onepress@plos.org.

Kind regards,

Marcia B. Aguila, Ph.D.

Academic Editor

PLOS One

Additional Editor Comments (optional):

Reviewers' comments:

Reviewer’s Responses to Questions

**Comments to the Author**

Reviewer #2: All comments have been addressed

**********

2. Is the manuscript technically sound, and do the data support the conclusions?

Reviewer #2: Yes

**********

3. Has the statistical analysis been performed appropriately and rigorously?

Reviewer #2: Yes

**********

4. Have the authors made all data underlying the findings in their manuscript fully available?

The PLOS Data policy

Reviewer #2: Yes

**********

5. Is the manuscript presented in an intelligible fashion and written in standard English?

Reviewer #2: Yes

**********

Reviewer #2: PONE-D-26-20026R3 - Ketogenic diet–induced changes in hepatic drug metabolism with potential implications for ozanimod pharmacokinetics in mice

The authors have satisfactorily addressed the remaining concerns raised in my previous review. In particular, the pharmacokinetic findings are now consistently presented as a non-significant trend toward increased exposure to the parent compound, and the limitations arising from the absence of active-metabolite measurements are clearly acknowledged. The interpretation of measurements obtained from pooled samples has also been appropriately restrained, and the proposed relationships among ketogenic diet, gut microbiota, inflammatory changes, CYP activity, and ozanimod disposition are now framed as hypotheses rather than established causal mechanisms.

The revised Discussion appropriately recognizes the exploratory nature of the pharmacokinetic findings and the limitations of the experimental design. I do not identify any remaining issue that would warrant an additional round of revision.

**********

what does this mean?). If published, this will include your full peer review and any attached files.

If you choose “no”, your identity will remain anonymous but your review may still be made public.

**Do you want your identity to be public for this peer review?**  For information about this choice, including consent withdrawal, please see our Privacy Policy

Reviewer #2: **Yes:** Carlos Alberto Mandarim-de-Lacerda

**********

# Editor report: Acceptance letter

**DOI:** 10.1371/journal.pone.0357797.r008

**Authors:** Marcia Aguila

PONE-D-26-20026R3

PLOS One

Dear Dr. Jourová,

I'm pleased to inform you that your manuscript has been deemed suitable for publication in PLOS One. Congratulations! Your manuscript is now being handed over to our production team.

At this stage, our production department will prepare your paper for publication. This includes ensuring the following:

* All references, tables, and figures are properly cited

* All relevant supporting information is included in the manuscript submission,

* There are no issues that prevent the paper from being properly typeset

You will receive further instructions from the production team, including instructions on how to review your proof when it is ready. Please keep in mind that we are working through a large volume of accepted articles, so please give us a few days to review your paper and let you know the next and final steps.

Lastly, if your institution or institutions have a press office, please let them know about your upcoming paper now to help maximize its impact. If they'll be preparing press materials, please inform our press team within the next 48 hours. Your manuscript will remain under strict press embargo until 2 pm Eastern Time on the date of publication. For more information, please contact onepress@plos.org.

You will receive an invoice from PLOS for your publication fee after your manuscript has reached the completed accept phase. If you receive an email requesting payment before acceptance or for any other service, this may be a phishing scheme. Learn how to identify phishing emails and protect your accounts at https://explore.plos.org/phishing.

If we can help with anything else, please email us at customercare@plos.org.

Thank you for submitting your work to PLOS One and supporting open access.

Kind regards,

PLOS One Editorial Office Staff

on behalf of

Dr. Marcia B. Aguila

Academic Editor

PLOS One
